# Improved rough approximations based on variable *J*-containment neighborhoods

**DOI:** 10.1007/s41066-023-00379-w

**Published:** 2023-04-14

**Authors:** Tingting Zheng

**Affiliations:** School of Mathematical Sciences, Anhui University, 111 Jiulong Road, Hefei, 230601 Anhui People’s Republic of China

**Keywords:** Variable *j*-containment neighborhood ($V_{j}^{\beta }$-neighborhood), Lower and upper approximation, Attribute reduction, Topology structure, Accuracy measure, Dependence measure

## Abstract

Classic generalized rough set model in neighborhood systems provides a more general framework for depicting approximations, while it may meet the non-reflexive situations. Some scholars put forward different neighborhoods, such as adhesion neighborhoods (briefly, $P_{j}$-neighborhoods), containment neighborhoods (briefly, $C_{j}$-neighborhoods), and $E_{j}$-neighborhoods. However, not all of them are reflexive. Moreover, the granularity of $P_{j}$-neighborhoods and $C_{j}$-neighborhoods are too fine, and that of $E_{j}$-neighborhoods too coarse. To solve the problem, we aim to design a novel construction approach of neighborhoods, called variable *j*-containment neighborhoods (briefly, $V_{j}^{\beta }$-neighborhoods), which satisfies the reflexivity and the granularity so flexible that the neighborhood space can adjust the granularity to meet the needs of problems. We generalize three kinds of rough approximations in $V_{j}^{\beta }$-neighborhood spaces and discuss their properties. What’s more, we analyze the topology structures relying on $V_{j}^{\beta }$-neighborhood spaces and compare our proposed approach with the existing approaches. By selecting the appropriate parameter $\beta$, our neighborhood system is more flexible in adjusting the granularity to fit problem requirements. And illustrative examples demonstrate the advantages of the proposed rough set model to attribute reduction in incomplete information systems.

## Introduction

Rough set theory, proposed by Pawlak (1991), described as a pair of approximations, is a useful tool associated with granular computing for manipulating incomplete, vague, and imperfect knowledge. It has been applied to many fields of science and engineering, such as data mining, imaging process, medical diagnosis, oil extraction, and so on. The classical rough set model is built in equivalence relations. Based on equivalence classes, the uncertain knowledge is evaluated by the exact lower and upper approximations.

However, the indiscernibility relation is too restrictive to extend the applications of rough set theory. Some researchers focus on the relations in the fuzzy environment. Akram et al. (2020, 2021) studied the accuracy measures of fuzzy indiscernibility relations to extract granular structures. Other researchers try to define rough set models based on neighborhood systems rather than based on some relations.  Lin (1997) proposed the concept of a general neighborhood system, as a “finite type” topology, which can express some negligible uncertainty effectively.  Yao (1998) introduced a framework for the formulation, interpretation, and comparison of neighborhood systems and gave the relation between generalizing rough sets and neighborhood-based rough sets.  Lin (1998a, 1998b) and  EI-Bably et al. (2022) discussed the general topology in generalized neighborhood rough sets. Wu and Zhang (2002) systemically studied six classes of *k*-step neighborhood systems based on binary relations in a finite universe. Lately, Akram et al. (2023) provided rough Pythagorean fuzzy set models in *k*-step neighborhood systems. Yang et al. (2011) improved the accuracy of approximation using the coverings induced by maximal consistent blocks in incomplete information systems. Furthermore, Wang et al. (2018) proposed the notion of local neighborhood rough sets, motivated by local rough sets (Qian et al. 2018), to analyze big data using a semi-supervised approach with limited labeled data. Recently,  Trivedi and Ramanna (2021) developed a hybrid geometry method with Voronoi diagrams and tolerance-based neighborhoods. These works make neighborhood rough sets more realistic and useful.

In practical applications, due to the missing of acquiring data, the uncertainty values of data characters, and the limitation of some human factors, the information systems are often incomplete. We cannot be sure whether an element must belong to the neighborhood of an element. And sometimes, it is possible to encounter that a neighborhood is not reflexive, such that the lower and upper approximations cannot include the target subsets. Abd El-Monsef et al. (2014) proposed the eight types of $N_{j}$-neighborhood, and Atef et al. (2020) derived the three types of rough sets in $N_{j}$-neighborhood spaces, where $j=1,2,\cdots ,8$. Herein, $N_{1}$-neighborhoods and $N_{2}$-neighborhoods can be regarded as classic right and left neighborhoods induced by any binary relations, respectively, while the other six $N_{j}$-neighborhoods are the extensions of $N_{1}$ or $N_{2}$-neighborhoods. And EI Atik et al. (2021) used $N_{j}$-neighborhoods to approximate graphs. Furthermore, Atef et al. (2020, 2022) presented the three types of rough set models in adhesion neighborhood (briefly, $P_{j}$-neighborhood) spaces. The $P_{j}$-neighborhood rough sets can increase the accuracy measure and reduce the boundary regions of subsets. Al-shami (2021) put forward three rough set models based on $E_{j}$-neighborhoods and constructed rough approximations induced by $E_{j}$ topology. And  Al-shami et al. (2021) introduced the concept of containment neighborhoods (briefly, $C_{j}$-neighborhoods) and improved the accuracy of rough set models induced by $C_{j}$-neighborhoods comparing with $N_{j}$, $E_{j}$-neighborhoods. And these new models are used for COVID-19 medical diagnosis.

However, it should be noted that the above neighborhood systems may have some limitations in practical applications. First, for example, let $U=\{x_{1},x_{2},x_{3}\}$ and $B=\{(x_{1}, x_{2}),(x_{2}, x_{1}), (x_{2},x_{2}), (x_{2},x_{3}), (x_{3},x_{3})\}$ be a binary relation on *U* and $N_{1}(x)=\{y\in U \mid (x,y)\in B \}$ be the $N_{1}$-neighborhood of an element $x\in U$, defined by  Atef et al. (2022). Then, $N_{1}(x_{1})=\{x_{2}\}$ which shows $N_{1}$-neighborhood systems may not satisfy the reflexivity. Second,  Atef et al. (2022), Al-shami (2021) and Al-shami et al. (2021) gave the following concepts:

$$\begin{aligned} P_j (x)&= \{ y\in U \vert N_j (y) = N_j (x) \},\\ C_j (x)&= \{ y\in U \vert N_j (y)\subseteq N_j (x) \}, \\ E_j (x)&= \{ y\in U \vert N_j (y)\cap N_j (x)\ne \emptyset \}. \end{aligned}$$

Here, $j=1,2,3,4$. Obviously that $C_{j}(x)\subseteq E_{j}(x)$. And if the binary relation inducing $N_{j}$-neighborhoods is serial, and $P_{j}(x)\subseteq C_{j}(x)$. It is easy to notice that both $P_{j}$ and $C_{j}$-neighborhoods’ constrained characteristics are too harsh, while that of $E_{j}$-neighborhoods are too loose to adapt to most situations. That results in the granularity of $P_{j}$ and $C_{j}$-neighborhoods being too fine and that of $E_{j}$-neighborhoods being too coarse.

To solve the problem, we introduce a parameter $\beta$ as a threshold of the inclusion degrees and propose the notion of variable *j*-containment neighborhoods (briefly, $V_{j}^{\beta }$-neighborhoods). For incomplete information systems, the rough set model based on $V_{j}^{\beta }$-neighborhoods allows a flexible neighborhood region by a precision variable.

The motivation of this paper includes: (1) How to avoid non-reflexive neighborhoods? (2) How to build the novel neighborhood systems such that whose granularity is coarser than $E_{j}$-neighborhood systems’ and finer than $C_{j}$-neighborhood systems’? (2) How to construct the approximations based on the novel neighborhoods? (3) What is the topological structure of the novel neighborhood spaces? (4) How to select the proper granularity for facing real data mining?

The paper is organized as follows. Section 2 recalls some basic concepts of neighborhood systems, rough sets based on generalized neighborhoods, and general topology. In Sect. 3, we analyze the problem of the existing generalized constructed neighborhood systems and introduce the concept of $V_{j}^{\beta }$-neighborhood according to variable inclusion degrees, and discuss the properties of the novel neighborhood. Furthermore, we define the three pairs of approximation sets in $V_{j}^{\beta }$-neighborhood spaces and deduce the relationship among them in Sect. 4. Section 5 analyzes the topology structure of $V_{j}^{\beta }$-neighborhood spaces and discusses the interior and closure operators in the novel spaces. And Sect. 6 shows the application of rough approximations based on $V_{j}^{\beta }$-neighborhoods. Finally, we give some conclusions and make a plan for future research in the last section.

## Preliminaries

Throughout this paper, let *U* be a nonempty finite universe.

### Binary relation and neighborhood

#### Definition 1

(Lin 1998a; Yao 1998) A binary relation *B* on *U* is a subset of the Cartesian product $U\times U$. For any $x\in U$, the binary neighborhood of *x* is defined by $B_{s}(x)=\{y\in U\vert (x,y)\in B\}.$

#### Definition 2

(Lin 1998a; Yao 1998) A binary relation *B* on *U* is said to be:

(i) reflexive if for any $x\in U$, $x\in B_{s}(x)$;
(ii) symmetric if for any $x,y\in U$, $y\in B_{s}(x)\Rightarrow x\in B_{s}(y)$;
(iii) transitive if for any $x,y,z\in U$, $y\in B_{s}(x),~z\in B_{s}(y)\Rightarrow z\in B_{s}(x)$;
(iv) equivalent if it is reflexive, symmetric, and transitive ;
(v) serial if for any $x\in U$, there exists a $y\in U$, such that $y\in B_{s}(x)$;
(vi) inverse serial if for any $x\in U$, there exists a $y\in U$, such that $x\in B_{s}(y)$;
(vii) Euclidean, if for any $x,y,z\in U$, $y\in B_{s}(x), z\in B_{s}(x)\Rightarrow z\in B_{s}(y)$.

### Rough set based on existing constructed neighborhoods

Abd El-Monsef et al. (2014) first proposed the notions of *j*-neighborhood of an element, and Atef et al. (2020, 2022) and Al-shami et al. (2021, 2021) generalized them to the following other three kinds of constructed neighborhoods. They tried to construct rough sets in these new neighborhood spaces.

#### Definition 3

(Abd EI-Monsef et al. 2014; Atef et al. 2020) Let *B* be a binary relation on *U* and $x\in U$. The $N_{j}$-neighborhoods of *x* (denoted by $N_{j}(x)$) are defined as follows ($j=1,2,\cdots ,8$):

(i) $$N_{1}(x)=\{y\in U\vert y\in B_{s}(x)\}$$;
(ii) $$N_{2}(x)=\{y\in U\vert x\in B_{s}(y)\}$$;
(iii) $$N_{3}(x)=\left\{ \begin{aligned}&\bigcap \limits _{x\in N_{1}(y)}N_{1}(y),~&\{y\in U\vert x\in N_{1}(y)\}\ne \emptyset ;\\ {}&\emptyset ,~&\{y\in U\vert x\in N_{1}(y)\}=\emptyset ;\end{aligned}\right.$$
(iv) $$N_{4}(x)=\left\{ \begin{aligned}&\bigcap \limits _{x\in N_{2}(y)}N_{2}(y),~&\{y\in U\vert x\in N_{2}(y)\}\ne \emptyset ;\\ {}&\emptyset ,~&\{y\in U\vert x\in N_{2}(y)\}=\emptyset ;\end{aligned}\right.$$
(v) $$N_{5}(x)=N_{1}(x)\cap N_{2}(x)$$;
(vi) $$N_{6}(x)=N_{1}(x)\cup N_{2}(x)$$;
(vii) $$N_{7}(x)=N_{3}(x)\cap N_{4}(x)$$;
(viii) $$N_{8}(x)=N_{3}(x)\cup N_{4}(x)$$.

Here, $N_{j}$ can be regarded as a map which associates each $x\in U$ with its $N_{j}$-neighborhood in $2^{U}$. The triple $(U,B,N_{j})$ is called an $N_{j}$-neighborhood space.

#### Definition 4

(Atef et al. 2020) Let $(U,B,N_{j})$ be an $N_{j}$-neighborhood space ($j=1,2,\cdots ,8$) and $X\subseteq U$. The pair $\langle \underline{B_{N_{j}}}(X),\overline{B_{N_{j}}}(X)\rangle$ of lower and upper approximation of *X* based on $N_{j}$-neighborhoods is defined by

$$\begin{aligned}& \underline{B_{N_{j}}}(X)=\{x\in U \mid N_{j}(x)\subseteq X\};\\&\overline{B_{N_{j}}}(X)=\{x\in U \mid N_{j}(x)\cap X \ne \emptyset \}. \end{aligned}$$

#### Definition 5

(Al-shami 2021; Atef et al. 2020) Let *B* be a binary relation on *U* and $x\in U$. The $P_{j}$-neighborhoods of *x* (denoted by $P_{j}(x)$) are defined as follows ($j=1,2,\cdots ,8$):

(i) $$P_{1}(x)=\{y\in U\vert N_{1}(y)=N_{1}(x)\};$$
(ii) $$P_{2}(x)=\{y\in U\vert N_{2}(y)=N_{2}(x)\};$$
(iii) $$P_{3}(x)=\{y\in U\vert N_{3}(y)=N_{3}(x)\};$$
(iv) $$P_{4}(x)=\{y\in U\vert N_{4}(y)=N_{4}(x)\};$$
(v) $$P_{5}(x)=P_{1}(x)\cap P_{2}(x);$$
(vi) $$P_{6}(x)=P_{1}(x)\cup P_{2}(x);$$
(vii) $$P_{7}(x)=P_{3}(x)\cap P_{4}(x);$$
(viii) $$P_{8}(x)=P_{3}(x)\cup P_{4}(x).$$

The triple $(U,B,P_{j})$ is called a $P_{j}$-neighborhood space.

#### Definition 6

(Atef et al. 2020) Let $(U,B,P_{j})$ be a $P_{j}$-neighborhood space ($j=1,2,\cdots ,8$) and $X\subseteq U$. The pair $\langle \underline{B_{P_{j}}}(X),\overline{B_{P_{j}}}(X)\rangle$ of lower and upper approximation of *X* based on $P_{j}$-neighborhoods is defined by

$$\begin{aligned} &\underline{B_{P_{j}}}(X)=\{x\in U \mid P_{j}(x)\subseteq X\};\\&\overline{B_{P_{j}}}(X)=\{x\in U \mid P_{j}(x)\cap X \ne \emptyset \}. \end{aligned}$$

#### Definition 7

(Al-shami et al. 2021) Let *B* be a binary relation on *U* and $x\in U$. The $E_{j}$-neighborhoods of *x* (denoted by $E_{j}(x)$) are defined as follows ($j=1,2,\cdots ,8$):

(i) $$E_{1}(x)=\{y\in U\vert N_{1}(y)\cap N_{1}(x)\ne \emptyset \};$$
(ii) $$E_{2}(x)=\{y\in U\vert N_{2}(y)\cap N_{2}(x)\ne \emptyset \};$$
(iii) $$E_{3}(x)=\{y\in U\vert N_{3}(y) \cap N_{3}(x)\ne \emptyset \};$$
(iv) $$E_{4}(x)=\{y\in U\vert N_{4}(y) \cap N_{4}(x)\ne \emptyset \};$$
(v) $$E_{5}(x)=E_{1}(x)\cap E_{2}(x);$$
(vi) $$E_{6}(x)=E_{1}(x)\cup E_{2}(x);$$
(vii) $$E_{7}(x)=E_{3}(x)\cap E_{4}(x).$$
(viii) $$E_{8}(x)=E_{3}(x)\cup E_{4}(x).$$

The triple $(U,B,E_{j})$ is called an $E_{j}$-neighborhood space.

#### Definition 8

(Al-shami et al. 2021) Let $(U,B,E_{j})$ be an $E_{j}$-neighborhood space ($j=1,2,\cdots ,8$) and $X\subseteq U$. The pair $\langle \underline{B_{E_{j}}}(X),\overline{B_{E_{j}}}(X)\rangle$ of lower and upper approximation of *X* based on $E_{j}$-neighborhoods is defined by

$$\begin{aligned} &\underline{B_{E_{j}}}(X)=\{x\in U \mid E_{j}(x)\subseteq X\};\\&\overline{B_{E_{j}}}(X)=\{x\in U \mid E_{j}(x)\cap X \ne \emptyset \}. \end{aligned}$$

#### Definition 9

(Al-shami 2021) Let *B* be a binary relation on *U* and $x\in U$. The $C_{j}$-neighborhoods of *x* (denoted by $C_{j}(x)$) are defined as follows ($j=1,2,\cdots ,8$):

(i) $$C_{1}(x)=\{y\in U\vert N_{1}(y)\subseteq N_{1}(x)\};$$
(ii) $$C_{2}(x)=\{y\in U\vert N_{2}(y)\subseteq N_{2}(x)\};$$
(iii) $$C_{3}(x)=\{y\in U\vert N_{3}(y) \subseteq N_{3}(x)\};$$
(iv) $$C_{4}(x)=\{y\in U\vert N_{4}(y) \subseteq N_{4}(x)\};$$
(v) $$C_{5}(x)=C_{1}(x)\cap C_{2}(x);$$
(vi) $$C_{6}(x)=C_{1}(x)\cup C_{2}(x);$$
(vii) $$C_{7}(x)=C_{3}(x)\cap C_{4}(x);$$
(viii) $$C_{8}(x)=C_{3}(x)\cup C_{4}(x).$$

The triple $(U,B,C_{j})$ is called a $C_{j}$-neighborhood space.

#### Definition 10

(Al-shami 2021) Let $(U,B,C_{j})$ be a $C_{j}$-neighborhood space ($j=1,2,\cdots ,8$) and $X\subseteq U$. The pair $\langle \underline{B_{C_{j}}}(X),\overline{B_{C_{j}}}(X)\rangle$ of lower and upper approximation of *X* based on $C_{j}$-neighborhoods is defined by

$$\begin{aligned} &\underline{B_{C_{j}}}(X)=\{x\in U \mid C_{j}(x)\subseteq X\};\\&\overline{B_{C_{j}}}(X)=\{x\in U \mid C_{j}(x)\cap X \ne \emptyset \}. \end{aligned}$$

### General topology

#### Definition 11

(Kelley 1995) Let ${\mathcal {U}}_{x}$ be the non-void family of all neighborhoods of *x*, here $x\in U$. Then:

(i) If $$X\in {\mathcal {U}}_{x}$$, then $$x\in X$$;
(ii) If $X,Y\in {\mathcal {U}}_{x}$, then $X \cap Y\in {\mathcal {U}}_{x}$;
(iii) If $X\in {\mathcal {U}}_{x}$ and $X\subseteq Y$, then $Y\in {\mathcal {U}}_{x}$;
(iv) If $X\in {\mathcal {U}}_{x}$, then there is a member *Y* of ${\mathcal {U}}_{x}$, such that $Y\subseteq X$ and $Y\in {\mathcal {U}}_{y}$ for each *y* in $Y$.

If all the family ${\mathcal {U}}_{x}$ satisfy (i), (ii), and (iii) for all *x* in *U*, then the family ${\mathcal {T}}$ of all sets *X*, such that $U\in {\mathcal {U}}_{x}$ whenever $x\in U$, is a topology of *U*. If (iv) is also satisfied, then ${\mathcal {U}}_{x}$ is the neighborhood system of *x* relative to the topology ${\mathcal {T}}$.

#### Definition 12

(Kelley 1995) Let *U* be a topology space, $x\in U$, and ${\mathcal {U}}_{x}$ be a neighborhood system of *x*. If the subset ${\mathcal {V}}_{x}$ of ${\mathcal {U}}_{x}$ satisfies every neighborhood of *x* contains a member of ${\mathcal {V}}_{x}$, then ${\mathcal {V}}_{x}$ is called a base of ${\mathcal {U}}_{x}$, or a local base at *x*.

Interior and closure operators are fundamental concepts in a topology space. Kuratowski’s axiom gives the characters of interior and closure operators.

#### Theorem 1

(Kelley 1995) Let $(U,{\mathcal {T}})$ be a topology space. A closure (or interior) operator $cl: 2^{U}\rightarrow 2^{U}$ (or $int: 2^{U}\rightarrow 2^{U})$ satisfies that for any $X,Y\subseteq U$, the following holds:

(i) $$cl(\emptyset )=\emptyset$$ (or $$int(U)=U)$$;
(ii) $$cl(X \cup Y)=cl(X) \cup cl(Y)$$ (or $$int(X \cap Y)=int(X) \cap int(Y))$$;
(iii) $$X\subseteq cl(X)$$ (or $$int(X)\subseteq X)$$;
(iv) $$cl(cl(X))=cl(X)$$ (or $$int(X)=int(int(X)))$$.

## Variable *j*-containment neighborhoods

### Existing neighborhood analysis

For $N_{j}$-neighborhood space, we can find $N_{1}$, $N_{2}$, $N_{5}$, and $N_{6}$-neighborhood operators are consistent with the successor, predecessor, predecessor-and-successor, and predecessor-or-successor neighborhoods presented by  Yao (1998), respectively. And the lower and upper approximations in Definition 4 can be seen as the rough sets in binary neighborhood system in  Lin (1998b) or 1-neighborhood systems (Yao 1998).

For $P_{j}$-neighborhood space, the eight $P_{j}$-neighborhood operators form the eight partitions on *U*. They transform the eight original spaces into the eight finest approximation spaces based on equivalence relations, respectively. Their equivalent classes are the finest granular, which can keep all the information of the original space, which can be seen as the core knowledge of their original spaces in Zheng (2020).

For $C_{j}$-neighborhood space, since $P_{j}(x)\subseteq C_{j}(x)$ for any *j* and any $x\in U$, the granular of $(U,B,C_{j})$ is coarser than that of $(U,B,P_{j})$ (Proposition 3.4 in  Al-shami (2021)). However, the condition is still too strict. Such as $C_{1}$-neighborhood, if $y\in C_{1}(x)$, then $N_{1}(y)\subseteq N_{1}(x)$. That means only those elements whose $N_{1}$-neighborhood is completely contained in $N_{1}(x)$ can enter the $C_{1}$-neighborhood of *x*.

For $E_{j}$-neighborhood space, take $E_{1}$-neighborhood as an example, although it relaxes the condition that is $N_{1}(y)\cap N_{1}(x)\ne \emptyset$, the neighborhood space may not cover the whole universe if there exists some neighborhood $N_{1}(x)=\emptyset$. And the granular of $(U, B, E_{j})$ is the coarsest among the existing neighborhood spaces.

Based on the above analysis, we try to construct a novel neighborhood with strict variable conditions and demonstrate flexibility in granularity selection.

### $V_{j}^{\beta }$-neighborhoods

This section will extend the above three generalized neighborhoods to variable containment neighborhoods based on inclusion degrees, which can hold the advantages of the above three neighborhoods. First, we define the inclusion degree of $N_{j}$-neighborhood spaces.

#### Definition 13

Let $(U,B,N_{j})$ be a $N_{j}$-neighborhood space. For $x,y\in U$, the inclusion degree of $N_{j}(x)$ with respect to $N_{j}(y)$ is defined as follows ($j=1,2,\cdots ,8$):

$$\begin{aligned} D(N_{j}(x)/ N_{j}(y))=\left\{ \begin{aligned}&\dfrac{\mid N_{j}(x)\cap N_{j}(y)\mid }{\mid N_{j}(y)\mid },~&N_{j}(y)\ne \emptyset ;\\ {}&1,~&N_{j}(y)=\emptyset .\end{aligned}\right. \end{aligned}$$

Here, $\vert \cdot \vert$ means the cardinality of the set.

#### Definition 14

Let *B* be a binary relation on *U*, $\beta \subseteq (0,1]$ and $x\in U$. The $V_{j}^{\beta }$-neighborhoods of *x* (denoted by $V_{j}^{\beta }(x)$) are defined as follows ($j=1,2,\cdots ,8$):

(i) $$V_{1}^{\beta }(x)=\{y\in U\vert D(N_{1}(x)/ N_{1}(y))\geqslant \beta \};$$
(ii) $$V_{2}^{\beta }(x)=\{y\in U\vert D(N_{2}(x)/ N_{2}(y))\geqslant \beta \};$$
(iii) $$V_{3}^{\beta }(x)=\{y\in U\vert D(N_{3}(x)/ N_{3}(y))\geqslant \beta \};$$
(iv) $$V_{4}^{\beta }(x)=\{y\in U\vert D(N_{4}(x)/ N_{4}(y))\geqslant \beta \};$$
(v) $$V_{5}^{\beta }(x)=V_{1}^{\beta }(x)\cap V_{2}^{\beta }(x);$$
(vi) $$V_{6}^{\beta }(x)=V_{1}^{\beta }(x)\cup V_{2}^{\beta }(x);$$
(vii) $$V_{7}^{\beta }(x)=V_{3}^{\beta }(x)\cap V_{4}^{\beta }(x);$$
(viii) $$V_{8}^{\beta }(x)=V_{3}^{\beta }(x)\cup V_{4}^{\beta }(x).$$

The triple $(U,B,V_{j}^{\beta })$ is called a $V_{j}^{\beta }$-neighborhood space.

#### Example 1

Let *B* be a binary relation on $U=\{x_{1},x_{2},x_{3},x_{4},x_{5}\}$, where $B=\{(x_{1},x_{1}),(x_{1},x_{2}),(x_{1},x_{3}),(x_{2},x_{3}),(x_{2},x_{4}),(x_{2},x_{5}),(x_{3},x_{3}),(x_{3},x_{5}),(x_{4},x_{3}),(x_{4},x_{4})\}$. Calculate all the $N_{j}$, $E_{j}$, $P_{j}$, $C_{j}$ and $V_{j}^{0.6}$-neighborhoods, $j=1,2,\cdots ,8$.

**Table 1:** $(U,B,N_{j})$ in Example 1

	$x_{1}$	$x_{2}$	$x_{3}$	$x_{4}$	$x_{5}$
$N_{1}$	$\{x_{1},x_{2},x_{3}\}$	$\{x_{3},x_{4},x_{5}\}$	$\{x_{3},x_{5}\}$	$\{x_{3},x_{4}\}$	$\emptyset$
$N_{2}$	$\{x_{1}\}$	$\{x_{1}\}$	$\{x_{1},x_{2},x_{3},x_{4}\}$	$\{x_{2},x_{4}\}$	$\{x_{2},x_{3}\}$
$N_{3}$	$\{x_{1},x_{2},x_{3}\}$	$\{x_{1},x_{2},x_{3}\}$	$\{x_{3}\}$	$\{x_{3},x_{4}\}$	$\{x_{3},x_{5}\}$
$N_{4}$	$\{x_{1}\}$	$\{x_{2}\}$	$\{x_{2},x_{3}\}$	$\{x_{2},x_{4}\}$	$\emptyset$
$N_{5}$	$\{x_{1}\}$	$\emptyset$	$\{x_{3}\}$	$\{x_{4}\}$	$\emptyset$
$N_{6}$	$\{x_{1},x_{2},x_{3}\}$	$\{x_{1},x_{3},x_{4},x_{5}\}$	*U*	$\{x_{2},x_{3},x_{4}\}$	$\{x_{2},x_{3}\}$
$N_{7}$	$\{x_{1}\}$	$\{x_{2}\}$	$\{x_{3}\}$	$\{x_{4}\}$	$\emptyset$
$N_{8}$	$\{x_{1},x_{2},x_{3}\}$	$\{x_{1},x_{2},x_{3}\}$	$\{x_{2},x_{3}\}$	$\{x_{2},x_{3},x_{4}\}$	$\{x_{3},x_{5}\}$

**Table 2:** $(U,B,E_{j})$ in Example 1

	$x_{1}$	$x_{2}$	$x_{3}$	$x_{4}$	$x_{5}$
$E_{1}$	$\{x_{1},x_{2},x_{3},x_{4}\}$	$\{x_{1},x_{2},x_{3},x_{4}\}$	$\{x_{1},x_{2},x_{3},x_{4}\}$	$\{x_{1},x_{2},x_{3},x_{4}\}$	$\emptyset$
$E_{2}$	$\{x_{1},x_{2},x_{3}\}$	$\{x_{1},x_{2},x_{3}\}$	*U*	$\{x_{3},x_{4},x_{5}\}$	$\{x_{3},x_{4},x_{5}\}$
$E_{3}$	*U*	*U*	*U*	*U*	*U*
$E_{4}$	$\{x_{1}\}$	$\{x_{2},x_{3},x_{4}\}$	$\{x_{2},x_{3},x_{4}\}$	$\{x_{2},x_{3},x_{4}\}$	$\emptyset$
$E_{5}$	$\{x_{1},x_{2},x_{3}\}$	$\{x_{1},x_{2},x_{3}\}$	$\{x_{1},x_{2},x_{3},x_{4}\}$	$\{x_{3},x_{4}\}$	$\emptyset$
$E_{6}$	$\{x_{1},x_{2},x_{3},x_{4}\}$	$\{x_{1},x_{2},x_{3},x_{4}\}$	*U*	*U*	$\{x_{3},x_{4},x_{5}\}$
$E_{7}$	$\{x_{1}\}$	$\{x_{2},x_{3},x_{4}\}$	$\{x_{2},x_{3},x_{4}\}$	$\{x_{2},x_{3},x_{4}\}$	$\emptyset$
$E_{8}$	*U*	*U*	*U*	*U*	*U*

**Table 3:** $(U,B,P_{j})$ in Example 1

	$x_{1}$	$x_{2}$	$x_{3}$	$x_{4}$	$x_{5}$
$P_{1}$	$\{x_{1}\}$	$\{x_{2}\}$	$\{x_{3}\}$	$\{x_{4}\}$	$\{x_{5}\}$
$P_{2}$	$\{x_{1},x_{2}\}$	$\{x_{1},x_{2}\}$	$\{x_{3}\}$	$\{x_{4}\}$	$\{x_{5}\}$
$P_{3}$	$\{x_{1},x_{2}\}$	$\{x_{1},x_{2}\}$	$\{x_{3}\}$	$\{x_{4}\}$	$\{x_{5}\}$
$P_{4}$	$\{x_{1}\}$	$\{x_{2}\}$	$\{x_{3}\}$	$\{x_{4}\}$	$\{x_{5}\}$
$P_{5}$	$\{x_{1}\}$	$\{x_{2}\}$	$\{x_{3}\}$	$\{x_{4}\}$	$\{x_{5}\}$
$P_{6}$	$\{x_{1},x_{2}\}$	$\{x_{1},x_{2}\}$	$\{x_{3}\}$	$\{x_{4}\}$	$\{x_{5}\}$
$P_{7}$	$\{x_{1}\}$	$\{x_{2}\}$	$\{x_{3}\}$	$\{x_{4}\}$	$\{x_{5}\}$
$P_{8}$	$\{x_{1},x_{2}\}$	$\{x_{1},x_{2}\}$	$\{x_{3}\}$	$\{x_{4}\}$	$\{x_{5}\}$

**Table 4:** $(U,B,C_{j})$ in Example 1

	$x_{1}$	$x_{2}$	$x_{3}$	$x_{4}$	$x_{5}$
$C_{1}$	$\{x_{1},x_{5}\}$	$\{x_{2},x_{3},x_{4},x_{5}\}$	$\{x_{3},x_{5}\}$	$\{x_{4},x_{5}\}$	$\{x_{5}\}$
$C_{2}$	$\{x_{1},x_{2}\}$	$\{x_{1},x_{2}\}$	*U*	$\{x_{4}\}$	$\{x_{5}\}$
$C_{3}$	$\{x_{1},x_{2},x_{3}\}$	$\{x_{1},x_{2},x_{3}\}$	$\{x_{3}\}$	$\{x_{3},x_{4}\}$	$\{x_{3},x_{5}\}$
$C_{4}$	$\{x_{1},x_{5}\}$	$\{x_{2},x_{5}\}$	$\{x_{2},x_{3},x_{5}\}$	$\{x_{2},x_{4},x_{5}\}$	$\{x_{5}\}$
$C_{5}$	$\{x_{1}\}$	$\{x_{2}\}$	$\{x_{3},x_{5}\}$	$\{x_{4}\}$	$\{x_{5}\}$
$C_{6}$	$\{x_{1},x_{2},x_{5}\}$	*U*	*U*	$\{x_{4},x_{5}\}$	$\{x_{5}\}$
$C_{7}$	$\{x_{1}\}$	$\{x_{2}\}$	$\{x_{3}\}$	$\{x_{4}\}$	$\{x_{5}\}$
$C_{8}$	$\{x_{1},x_{2},x_{3},x_{5}\}$	$\{x_{1},x_{2},x_{3},x_{5}\}$	$\{x_{2},x_{3},x_{5}\}$	$\{x_{2},x_{3},x_{4},x_{5}\}$	$\{x_{3},x_{5}\}$

**Table 5:** $(U,B,V_{j}^{0.6})$ in Example 1

	$x_{1}$	$x_{2}$	$x_{3}$	$x_{4}$	$x_{5}$
$V_{1}^{0.6}$	$\{x_{1},x_{5}\}$	$\{x_{2},x_{3},x_{4},x_{5}\}$	$\{x_{2},x_{3},x_{5}\}$	$\{x_{2},x_{4},x_{5}\}$	$\{x_{5}\}$
$V_{2}^{0.6}$	$\{x_{1},x_{2}\}$	$\{x_{1},x_{2}\}$	*U*	$\{x_{4}\}$	$\{x_{5}\}$
$V_{3}^{0.6}$	$\{x_{1},x_{2},x_{3}\}$	$\{x_{1},x_{2},x_{3}\}$	$\{x_{3}\}$	$\{x_{3},x_{4}\}$	$\{x_{3},x_{5}\}$
$V_{4}^{0.6}$	$\{x_{1},x_{5}\}$	$\{x_{2},x_{5}\}$	$\{x_{2},x_{3},x_{5}\}$	$\{x_{2},x_{4},x_{5}\}$	$\{x_{5}\}$
$V_{5}^{0.6}$	$\{x_{1}\}$	$\{x_{2}\}$	$\{x_{2},x_{3},x_{5}\}$	$\{x_{4}\}$	$\{x_{5}\}$
$V_{6}^{0.6}$	$\{x_{1},x_{2},x_{5}\}$	*U*	*U*	$\{x_{2},x_{4},x_{5}\}$	$\{x_{5}\}$
$V_{7}^{0.6}$	$\{x_{1}\}$	$\{x_{2}\}$	$\{x_{3}\}$	$\{x_{4}\}$	$\{x_{5}\}$
$V_{8}^{0.6}$	$\{x_{1},x_{2},x_{3},x_{5}\}$	$\{x_{1},x_{2},x_{3},x_{5}\}$	$\{x_{2},x_{3},x_{5}\}$	$\{x_{2},x_{3},x_{4},x_{5}\}$	$\{x_{3},x_{5}\}$

Tables 1, 2, 3, 4 and 5 show all the neighborhood spaces. We can find $N_{j}$ and $E_{j}$-neighborhood spaces which show some empty neighborhoods of some objects, $P_{j}$-neighborhoods of five objects are almost all single point sets, while the granular of $C_{j}$-neighborhoods and $V_{j}^{0.6}$-neighborhoods are relatively moderate.

#### Proposition 2

Any $V_{j}^{\beta }$-neighborhood space satisfies the following properties: For any $x,y\in U$, any $j=1,2,\cdots ,8$, and $\beta , \beta _{1}, \beta _{2} \in (0,1]$

(i) $$x\in V_{j}^{\beta }(x)$$.
(ii) $$V_{5}^{\beta }(x)\subseteq V_{1}^{\beta }(x)\subseteq V_{6}^{\beta }(x)$$; $$V_{5}^{\beta }(x)\subseteq V_{2}^{\beta }(x)\subseteq V_{6}^{\beta }(x)$$;      $$V_{7}^{\beta }(x)\subseteq V_{3}^{\beta }(x)\subseteq V_{8}^{\beta }(x)$$; $$V_{7}^{\beta }(x)\subseteq V_{4}^{\beta }(x)\subseteq V_{8}^{\beta }(x)$$.
(iii) If $N_{j}(y)\subseteq N_{j}(x)$, then $y\in V_{j}^{\beta }(x)$.
(iv) If $\beta _{1}<\beta _{2}$, then $V_{j}^{\beta _{2}}(x)\subseteq V_{j}^{\beta _{1}}(x)$.
(v) If $B$ is symmetric, then $V_{1}^{\beta }(x)=V_{2}^{\beta }(x)=V_{5}^{\beta }(x)=V_{6}^{\beta }(x)$ and $V_{3}^{\beta }(x)=V_{4}^{\beta }(x)=V_{7}^{\beta }(x)=V_{8}^{\beta }(x)$.
(vi) If $B$ is reflexive and transitive, then $V_{1}^{\beta }(x)=V_{3}^{\beta }(x)$, $V_{2}^{\beta }(x)=V_{4}^{\beta }(x)$, $V_{5}^{\beta }(x)=V_{7}^{\beta }(x)$ and $V_{6}^{\beta }(x)=V_{8}^{\beta }(x)$.
(vii) If $B$ is equivalent, then $V_{1}^{\beta }(x)=V_{2}^{\beta }(x)=\cdots =V_{8}^{\beta }(x)$.

#### Proof

If these claims are true when $j=1,2,3,4$, they would also hold when $j=5,6,7,8$ based on Definition 14. Therefore, we only prove the cases of $j=1,2,3,4$.

(i) Since $D(N_{j}(x)/N_{j}(x))\equiv 1$ always hold whether $N_{j}(x)$ is empty or not, for any $j=1,2,3,4$. Therefore, $x\in V_{j}^{\beta }(x)$ for any $\beta$ and any *j*.
(ii) The proof is obvious based on Definition 14.
(iii) If $N_{j}(y)\subseteq N_{j}(x)$, $D(N_{j}(x)/N_{j}(y))\equiv 1$. Therefore, $y\in V_{j}^{\beta }(x)$ for any $j=1,2,3,4$. Thus, $y\in V_{j}^{\beta }(x)$ for any $\beta$ and any *j*.
(iv) Suppose $y\in V_{j}^{\beta _{2}}(x)$. Then, $D(N_{j}(x)/N_{j}(y))\geqslant \beta _{2}$. Since $\beta _{1}<\beta _{2}$, $D(N_{j}(x)/N_{j}(y))\geqslant \beta _{1}$. Thus, $y\in V_{j}^{\beta _{1}}(x)$ which means $V_{j}^{\beta _{2}}(x)\subseteq V_{j}^{\beta _{1}}(x)$.
(v) If $B$ is symmetric, $N_{1}(x)=N_{2}(x)$ and $N_{3}(x)=N_{4}(x)$ for any $x\in U$. Therefore, $N_{5}(x)=N_{1}(x)\cap N_{2}(x)=N_{1}(x)$ and $N_{6}(x)=N_{1}(x)\cup N_{2}(x)=N_{1}(x)$. Similarly, $N_{7}(x)=N_{8}(x)=N_{3}(x)=N_{4}(x)$. Thus, $V_{1}^{\beta }(x)=V_{2}^{\beta }(x)=V_{5}^{\beta }(x)=V_{6}^{\beta }(x)$ and $V_{3}^{\beta }(x)=V_{4}^{\beta }(x)=V_{7}^{\beta }(x)=V_{8}^{\beta }(x)$ for any $\beta$.
(vi) Since $B$ is reflexive, then $x\in N_{1}(x)$ for any $x\in U$. Therefore, $N_{1}(x)\supseteq \bigcap \nolimits _{x\in N_{1}(y)}N_{1}(y)\ne \emptyset$. That is, $N_{1}(x)\supseteq N_{3}(x)$. Let $y\in N_{1}(x)$. Then, for any $N_{1}(z)$ containing *x*, $y\in N_{1}(z)$, because *B* is transitive. Therefore, $y\in \bigcap \nolimits _{x\in N_{1}(z)}N_{1}(z)=N_{3}(x)$. That means $N_{1}(x)\subseteq N_{3}(x)$. Hence $N_{1}(x)= N_{3}(x)$. Consequently, $V_{1}^{\beta }(x)= V_{3}^{\beta }(x)$. Similarly, $V_{2}^{\beta }(x)= V_{4}^{\beta }(x)$, $V_{5}^{\beta }(x)=V_{7}^{\beta }(x)$ and $V_{6}^{\beta }(x)=V_{8}^{\beta }(x)$.
(vii) According to claims (v) and (vi), we prove the claim obviously. $\square$

#### Proposition 3

Any $V_{j}^{\beta }$-neighborhood space satisfies the following properties: For any $x\in U$, any $j=1,2,\cdots ,8$ and $\beta \in (0,1]$

(i) $P_{j}(x)\subseteq C_{j}(x) \subseteq V_{j}^{\beta }(x)$; $C_{j}(x)=V_{j}^{\beta }(x)$ if and only if $\beta =1$.
(ii) If $B$ is serial and inverse serial, then $V_{j}^{\beta }(x)\subseteq E_{j}(x)$.
(iii) If $B$ is reflexive, then $P_{j}(x)\subseteq C_{j}(x)\subseteq V_{j}^{\beta }(x)\subseteq E_{j}(x)$.
(iv) If $B$ is equivalent, then $P_{j}(x)=C_{j}(x)=V_{j}^{\beta }(x)=N_{j}(x)=E_{j}(x)$.

#### Proof

(i) First, we have $P_{j}(x)\subseteq C_{j}(x)$ in Theorem 2 of (Al-shami 2021). Let $y\in C_{j}(x)$ for any $j=1,2,3,4$. Then, $N_{j}(y)\subseteq N_{j}(x)$ implies that $D(N_{j}(x)/N_{j}(y))=1$. Therefore, $y\in V_{j}^{\beta }(x)$. Thus, $P_{j}(x)\subseteq C_{j}(x)\subseteq V_{j}^{\beta }(x)$ for any $j=1,2,\cdots ,8$.
(ii) Since *B* is serial and inverse serial, $N_{j}(x)\ne \emptyset$ for any $j=1,2,3,4$ and any $x\in U$. Let $y\in V_{j}^{\beta }(x)$. Then, $D(N_{j}(x)/N_{j}(y))\geqslant \beta >0$, which shows $N_{j}(x)\cap N_{j}(y)\ne \emptyset$. Therefore, $y\in E_{j}(x)$. That means $V_{j}^{\beta }(x)\subseteq E_{j}(x)$ for $j=1,2,3,4$. Furthermore, based on Definition 14, we have $V_{j}^{\beta }(x)\subseteq E_{j}(x)$ for any $j=1,2,\cdots ,8$.
(iii) If $B$ is reflexive, *B* must be serial and inverse serial. The conclusion is obviously true based on the above two claims.
(iv) If $B$ is equivalent, *B* must be reflexive. Therefore, $P_{j}(x)\subseteq C_{j}(x) \subseteq V_{j}^{\beta }(x)\subseteq E_{j}(x)$ and $P_{j}(x)\subseteq C_{j}(x) \subseteq N_{j}(x)\subseteq E_{j}(x)$ for any *j*. When *B* is equivalent, the proof of $P_{j}(x)=N_{j}=E_{j}(x)$ has been given in Theorem 1 of Al-shami et al. (2021) and $P_{j}(x)=C_{j}(x)$ has been proved in Proposition 3.4 of Al-shami (2021). Hence, we have $P_{j}(x)=C_{j}(x)=V_{j}^{\beta }(x)=N_{j}(x)=E_{j}(x)$. $\square$

#### Remark 1

If *B* is equivalent, all kinds of neighborhood operators are the same, including eight $P_{j}$-neighborhoods, eight $C_{j}$-neighborhoods, eight $V_{j}^{\beta }$-neighborhoods, eight $N_{j}$-neighborhoods, and eight $E_{j}$-neighborhoods for any $x\in U$.

#### Remark 2

In general, $N_{j}$-neighborhoods and $V_{j}^{\beta }$-neighborhoods have no partial ordering relation. Even if *B* is reflexive, their relationship is the same.

## Rough sets in $V_{j}^{\beta }$-neighborhood spaces

### Approximations based on $V_{j}^{\beta }$-neighborhoods

#### Definition 15

Let $(U,B,V_{j}^{\beta })$ be a $V_{j}^{\beta }$-neighborhood space ($j=1,2,\cdots ,8$) and $X\subseteq U$. The type-1 rough set based on $V_{j}^{\beta }$-neighborhoods of *X* is the pair $\langle \underline{B_{V_{j}^{\beta }}^{(1)}}(X),\overline{B_{V_{j}^{\beta }}^{(1)}}(X)\rangle$ composed by the following lower and upper approximations:

$$\begin{aligned} &\underline{B_{V_{j}^{\beta }}^{(1)}}(X)=\{x\in U \mid V_{j}^{\beta }(x)\subseteq X\};\\&\overline{B_{V_{j}^{\beta }}^{(1)}}(X)=\{x\in U \mid V_{j}^{\beta }(x)\cap X \ne \emptyset \}. \end{aligned}$$

#### Proposition 4

Let $(U,B,V_{j}^{\beta })$ be a $V_{j}^{\beta }$-neighborhood space($j=1,2,\cdots ,8$) and $X,Y\subseteq U$. The type-1 lower and upper approximations based on $V_{j}^{\beta }$-neighborhoods satisfy:

(i) $$\underline{B_{V_{j}^{\beta }}^{(1)}}(X)=(\overline{B_{V_{j}^{\beta }}^{(1)}}(X^{C}))^{C}$$; $$\overline{B_{V_{j}^{\beta }}^{(1)}}(X)=(\underline{B_{V_{j}^{\beta }}^{(1)}}(X^{C}))^{C}$$;
(ii) $$\underline{B_{V_{j}^{\beta }}^{(1)}}(U)=U$$; $$\underline{B_{V_{j}^{\beta }}^{(1)}}(\emptyset )=\emptyset$$; $$\overline{B_{V_{j}^{\beta }}^{(1)}}(U)=U$$; $$\overline{B_{V_{j}^{\beta }}^{(1)}}(\emptyset )=\emptyset$$;
(iii) $$\underline{B_{V_{j}^{\beta }}^{(1)}}(X)\subseteq X \subseteq \overline{B_{V_{j}^{\beta }}^{(1)}}(X)$$;
(iv) $$X\subseteq Y\Rightarrow \underline{B_{V_{j}^{\beta }}^{(1)}}(X)\subseteq \underline{B_{V_{j}^{\beta }}^{(1)}}(Y)$$, $$\overline{B_{V_{j}^{\beta }}^{(1)}}(X)\subseteq \overline{B_{V_{j}^{\beta }}^{(1)}}(Y)$$;
(v) $$\underline{B_{V_{j}^{\beta }}^{(1)}}(X\cap Y)=\underline{B_{V_{j}^{\beta }}^{(1)}}(X)\cap \underline{B_{V_{j}^{\beta }}^{(1)}}(Y)$$; $$\overline{B_{V_{j}^{\beta }}^{(1)}}(X\cup Y)=\overline{B_{V_{j}^{\beta }}^{(1)}}(X)\cup \overline{B_{V_{j}^{\beta }}^{(1)}}(Y)$$;
(vi) $$\underline{B_{V_{j}^{\beta }}^{(1)}}(X\cup Y)\supseteq \underline{B_{V_{j}^{\beta }}^{(1)}}(X)\cup \underline{B_{V_{j}^{\beta }}^{(1)}}(Y)$$; $$\overline{B_{V_{j}^{\beta }}^{(1)}}(X\cap Y)\subseteq \overline{B_{V_{j}^{\beta }}^{(1)}}(X)\cap \overline{B_{V_{j}^{\beta }}^{(1)}}(Y)$$.
(vii) $$\underline{B_{V_{j}^{\beta }}^{(1)}}(\underline{B_{V_{j}^{\beta }}^{(1)}}(X))\subseteq \underline{B_{V_{j}^{\beta }}^{(1)}}(X)$$; $$\overline{B_{V_{j}^{\beta }}^{(1)}}(X)\subseteq \overline{B_{V_{j}^{\beta }}^{(1)}}(\overline{B_{V_{j}^{\beta }}^{(1)}}(X))$$.

#### Proof

(i) $$y\in \underline{B_{V_{j}^{\beta }}^{(1)}}(X) \Longleftrightarrow V_{j}^{\beta }(y)\subseteq X \Longleftrightarrow V_{j}^{\beta }(y)\cap X^{C}=\emptyset \Longleftrightarrow y\not \in \overline{B_{V_{j}^{\beta }}^{(1)}}(X^{C}) \Longleftrightarrow y\in (\overline{B_{V_{j}^{\beta }}^{(1)}}(X^{C}))^{C}$$. Similarly, $$\overline{B_{V_{j}^{\beta }}^{(1)}}(X)=( \underline{B_{V_{j}^{\beta }}^{(1)}}(X^{C}))^{C}$$.

(ii) Since $V_{j}^{\beta }(x)\subseteq U$ for any $x\in U$, then $U\subseteq \underline{B_{V_{j}^{\beta }}^{(1)}}(U)$. Let $y\in \underline{B_{V_{j}^{\beta }}^{(1)}}(U)$. Then, $V_{j}^{\beta }(y)\subseteq U$. Since $y\in V_{j}^{\beta }(y)$, $y\in U$. Therefore, $\underline{B_{V_{j}^{\beta }}^{(1)}}(U)\subseteq U$. Hence, $\underline{B_{V_{j}^{\beta }}^{(1)}}(U)=U$.

Since $x\in V_{j}^{\beta }(x)$ for any $x\in U$. Then, $V_{j}^{\beta }(x)\ne \emptyset$. Therefore, $\underline{B_{V_{j}^{\beta }}^{(1)}}(\emptyset )=\emptyset$. The following two claims are proved similarly.

(iii) Let $y\in \underline{B_{V_{j}^{\beta }}^{(1)}}(X)$. Then, $V_{j}^{\beta }(y)\subseteq X$. Since $y\in V_{j}^{\beta }(y)$, $y\subseteq X$. Therefore, $\underline{B_{V_{j}^{\beta }}^{(1)}}(X)\subseteq X$.

Let $z\in X$. Since $z\in V_{j}^{\beta }(z)$, $V_{j}^{\beta }(z)\cap X\ne \emptyset$. That means $z\in \overline{B_{V_{j}^{\beta }}^{(1)}}(X)$. Therefore, $X\subseteq \overline{B_{V_{j}^{\beta }}^{(1)}}(X)$.

(iv) Since $X\subseteq Y$, then $\underline{B_{V_{j}^{\beta }}^{(1)}}(X)=\{x\in U\vert V_{j}^{\beta }(x)\subseteq X\}\subseteq \{x\in U\vert V_{j}^{\beta }(x)\subseteq Y\}=\underline{B_{V_{j}^{\beta }}^{(1)}}(Y)$. At the same time, $\overline{B_{V_{j}^{\beta }}^{(1)}}(X)\subseteq \overline{B_{V_{j}^{\beta }}^{(1)}}(Y)$.

(v) $y\in \underline{B_{V_{j}^{\beta }}^{(1)}}(X \cap Y) \Longleftrightarrow V_{j}^{\beta }(y)\subseteq X \cap Y \Longleftrightarrow V_{j}^{\beta }(y)\subseteq X$ and $V_{j}^{\beta }(y) \subseteq Y \Longleftrightarrow y\in \underline{B_{V_{j}^{\beta }}^{(1)}}(X)$ and $y\in \underline{B_{V_{j}^{\beta }}^{(1)}}(Y) \Longleftrightarrow y\in \underline{B_{V_{j}^{\beta }}^{(1)}}(X) \cap \underline{B_{V_{j}^{\beta }}^{(1)}}(Y)$. Hence, $\underline{B_{V_{j}^{\beta }}^{(1)}}(X\cap Y)=\underline{B_{V_{j}^{\beta }}^{(1)}}(X)\cap \underline{B_{V_{j}^{\beta }}^{(1)}}(Y)$. Similarly, $\overline{B_{V_{j}^{\beta }}^{(1)}}(X\cup Y)=\overline{B_{V_{j}^{\beta }}^{(1)}}(X)\cup \overline{B_{V_{j}^{\beta }}^{(1)}}(Y)$.

(vi) Let $y\in \underline{B_{V_{j}^{\beta }}^{(1)}}(X)\cup \underline{B_{V_{j}^{\beta }}^{(1)}}(Y)$. Then, $V_{j}^{\beta }(y)\subseteq X$ or $V_{j}^{\beta }(y)\subseteq Y$. So $V_{j}^{\beta }(y)\subseteq X\cup Y$. That means $y \in \underline{B_{V_{j}^{\beta }}^{(1)}}(X \cup Y)$. Hence, $\underline{B_{V_{j}^{\beta }}^{(1)}}(X)\cup \underline{B_{V_{j}^{\beta }}^{(1)}}(Y)\subseteq \underline{B_{V_{j}^{\beta }}^{(1)}}(X\cup Y)$. Similarly, $\overline{B_{V_{j}^{\beta }}^{(1)}}(X\cap Y)\subseteq \overline{B_{V_{j}^{\beta }}^{(1)}}(X)\cap \overline{B_{V_{j}^{\beta }}^{(1)}}(Y)$.

(vii) The claim is obviously true based on claim (iii). $\square$

However, type-1 approximations in $V_{j}^{\beta }$-neighborhood spaces still do not satisfy s ome properties. Example 2 illustrates them.

#### Example 2

For $(U,B,V_{j}^{0.6})$ in Example 1, the type-1 lower and upper approximations based on $V_{j}^{0.6}$-neighborhoods are given in Table 6.

**Table 6:** Type-1 rough approximations based on $V_{j}^{0.6}$-neighborhoods in Example 2

*X*	$\underline{B_{V_{1}^{0.6}}^{(1)}}(X)$	$\overline{B_{V_{1}^{0.6}}^{(1)}}(X)$	$\underline{B_{V_{2}^{0.6}}^{(1)}}(X)$	$\overline{B_{V_{2}^{0.6}}^{(1)}}(X)$	$\underline{B_{V_{3}^{0.6}}^{(1)}}(X)$	$\overline{B_{V_{3}^{0.6}}^{(1)}}(X)$
$\{x_{3}\}$	$\emptyset$	$\{x_{2},x_{3}\}$	$\emptyset$	$\{x_{3}\}$	$\{x_{3}\}$	*U*
$\{x_{4}\}$	$\emptyset$	$\{x_{2},x_{4}\}$	$\{x_{4}\}$	$\{x_{3},x_{4}\}$	$\emptyset$	$\{x_{4}\}$
$\{x_{2},x_{3}\}$	$\emptyset$	$\{x_{2},x_{3},x_{4}\}$	$\emptyset$	$\{x_{1},x_{2},x_{3}\}$	$\{x_{3}\}$	*U*
$\{x_{2},x_{5}\}$	$\{x_{5}\}$	*U*	$\{x_{5}\}$	$\{x_{1},x_{2},x_{3},x_{5}\}$	$\emptyset$	$\{x_{1},x_{2},x_{5}\}$
$\{x_{3},x_{4}\}$	$\emptyset$	$\{x_{2},x_{3},x_{4}\}$	$\{x_{4}\}$	$\{x_{3},x_{4}\}$	$\{x_{3},x_{4}\}$	*U*
$\{x_{3},x_{5}\}$	$\{x_{5}\}$	*U*	$\{x_{3},x_{5}\}$	$\{x_{3},x_{5}\}$	$\{x_{3},x_{5}\}$	*U*
$\{x_{2},x_{3},x_{4}\}$	$\emptyset$	$\{x_{2},x_{3},x_{4}\}$	$\{x_{4}\}$	$\{x_{1},x_{2},x_{3},x_{4}\}$	$\{x_{3},x_{4}\}$	*U*
$\{x_{2},x_{3},x_{5}\}$	$\{x_{3},x_{5}\}$	*U*	$\{x_{5}\}$	$\{x_{1},x_{2},x_{3},x_{5}\}$	$\{x_{3},x_{5}\}$	*U*
$\{x_{2},x_{4},x_{5}\}$	$\{x_{4},x_{5}\}$	*U*	$\{x_{4},x_{5}\}$	*U*	$\emptyset$	$\{x_{1},x_{2},x_{4},x_{5}\}$

*X*	$\underline{B_{V_{4}^{0.6}}^{(1)}}(X)$	$\overline{B_{V_{4}^{0.6}}^{(1)}}(X)$	$\underline{B_{V_{5}^{0.6}}^{(1)}}(X)$	$\overline{B_{V_{5}^{0.6}}^{(1)}}(X)$	$\underline{B_{V_{6}^{0.6}}^{(1)}}(X)$	$\overline{B_{V_{6}^{0.6}}^{(1)}}(X)$
$\{x_{3}\}$	$\emptyset$	$\{x_{3}\}$	$\emptyset$	$\{x_{3}\}$	$\emptyset$	$\{x_{2},x_{3}\}$
$\{x_{4}\}$	$\emptyset$	$\{x_{4}\}$	$\{x_{4}\}$	$\{x_{4}\}$	$\emptyset$	$\{x_{2},x_{3},x_{4}\}$
$\{x_{2},x_{3}\}$	$\emptyset$	$\{x_{2},x_{3},x_{4}\}$	$\{x_{2}\}$	$\{x_{2},x_{3}\}$	$\emptyset$	$\{x_{1},x_{2},x_{3},x_{4}\}$
$\{x_{2},x_{5}\}$	$\{x_{2},x_{5}\}$	*U*	$\{x_{2},x_{5}\}$	$\{x_{2},x_{3},x_{5}\}$	$\{x_{5}\}$	*U*
$\{x_{3},x_{4}\}$	$\emptyset$	$\{x_{3},x_{4}\}$	$\{x_{4}\}$	$\{x_{3},x_{4}\}$	$\emptyset$	$\{x_{2},x_{3},x_{4}\}$
$\{x_{3},x_{5}\}$	$\{x_{5}\}$	*U*	$\{x_{5}\}$	$\{x_{3},x_{5}\}$	$\{x_{5}\}$	*U*
$\{x_{2},x_{3},x_{4}\}$	$\emptyset$	$\{x_{2},x_{3},x_{4}\}$	$\{x_{2},x_{4}\}$	$\{x_{2},x_{3},x_{4}\}$	$\emptyset$	$\{x_{1},x_{2},x_{3},x_{4}\}$
$\{x_{2},x_{3},x_{5}\}$	$\{x_{2},x_{3},x_{5}\}$	*U*	$\{x_{2},x_{3},x_{5}\}$	$\{x_{2},x_{3},x_{5}\}$	$\{x_{5}\}$	*U*
$\{x_{2},x_{4},x_{5}\}$	$\{x_{2},x_{4},x_{5}\}$	*U*	$\{x_{2},x_{4},x_{5}\}$	$\{x_{2},x_{3},x_{4},x_{5}\}$	$\{x_{4},x_{5}\}$	*U*

*X*	$\underline{B_{V_{7}^{0.6}}^{(1)}}(X)$	$\overline{B_{V_{7}^{0.6}}^{(1)}}(X)$	$\underline{B_{V_{8}^{0.6}}^{(1)}}(X)$	$\overline{B_{V_{8}^{0.6}}^{(1)}}(X)$		
$\{x_{3}\}$	$\{x_{3}\}$	$\{x_{3}\}$	$\emptyset$	*U*		
$\{x_{4}\}$	$\{x_{4}\}$	$\{x_{4}\}$	$\emptyset$	$\{x_{4}\}$		
$\{x_{2},x_{3}\}$	$\{x_{2},x_{3}\}$	$\{x_{2},x_{3}\}$	$\emptyset$	*U*		
$\{x_{2},x_{5}\}$	$\{x_{2},x_{5}\}$	$\{x_{2},x_{5}\}$	$\emptyset$	*U*		
$\{x_{3},x_{4}\}$	$\{x_{3},x_{4}\}$	$\{x_{3},x_{4}\}$	$\emptyset$	*U*		
$\{x_{3},x_{5}\}$	$\{x_{3},x_{5}\}$	$\{x_{3},x_{5}\}$	$\{x_{5}\}$	*U*		
$\{x_{2},x_{3},x_{4}\}$	$\{x_{2},x_{3},x_{4}\}$	$\{x_{2},x_{3},x_{4}\}$	$\emptyset$	*U*		
$\{x_{2},x_{3},x_{5}\}$	$\{x_{2},x_{3},x_{5}\}$	$\{x_{2},x_{3},x_{5}\}$	$\{x_{3},x_{5}\}$	*U*		
$\{x_{2},x_{4},x_{5}\}$	$\{x_{2},x_{4},x_{5}\}$	$\{x_{2},x_{4},x_{5}\}$	$\emptyset$	*U*		

From the example, we find several properties of some binary approximations may evaporate for $V_{j}^{\beta }$-neighborhood approximations.

For instance, for $X=\{x_{3}\}$, $\overline{B_{V_{1}^{0.6}}^{(1)}}(X)=\{x_{2},x_{3}\}$ and $\overline{B_{V_{1}^{0.6}}^{(1)}}(\{x_{2},x_{3}\})=\{x_{2},x_{3},x_{4}\}$, so $\overline{B_{V_{1}^{0.6}}^{(1)}}(X)\ne \overline{B_{V_{1}^{0.6}}^{(1)}}(\overline{B_{V_{1}^{0.6}}^{(1)}}(X))$; another case is that for $Y=\{x_{2},x_{3},x_{5}\}$, $\underline{B_{V_{8}^{0.6}}^{(1)}}(Y)=\{x_{3},x_{5}\}$ and $\underline{B_{V_{8}^{0.6}}^{(1)}}(\{x_{3},x_{5}\})=\{x_{5}\}$, so $\underline{B_{V_{8}^{0.6}}^{(1)}}(\underline{B_{V_{8}^{0.6}}^{(1)}}(Y))\ne \underline{B_{V_{8}^{0.6}}^{(1)}}(Y)$; besides, for $Z=\{x_{2},x_{5}\}$, $\underline{B_{V_{4}^{0.6}}^{(1)}}(Z)=\{x_{2},x_{5}\}$ and $\overline{B_{V_{4}^{0.6}}^{(1)}}(\{x_{2},x_{5}\})=U$, so $\overline{B_{V_{4}^{0.6}}^{(1)}}(\underline{B_{V_{4}^{0.6}}^{(1)}}(Z))\not \subseteq Z$; and for $T=\{x_{2},x_{3}\}$, $\overline{B_{V_{5}^{0.6}}^{(1)}}(T)=\{x_{2},x_{3}\}$ and $\underline{B_{V_{5}^{0.6}}^{(1)}}(\{x_{2},x_{3}\})=\{x_{2}\}$, so $T\not \subseteq \underline{B_{V_{5}^{0.6}}^{(1)}}(\overline{B_{V_{5}^{0.6}}^{(1)}}(T))$.

#### Proposition 5

Let $(U,B,V_{j}^{\beta })$ be a $V_{j}^{\beta }$-neighborhood space $(j=1,2,\cdots ,8)$ and $X\subseteq U$. The eight type-1 rough sets based on $V_{j}^{\beta }$-neighborhoods satisfy the following properties:

(i) $$\underline{B_{V_{6}^{\beta }}^{(1)}}(X) \subseteq \underline{B_{V_{1}^{\beta }}^{(1)}}(X)\subseteq \underline{B_{V_{5}^{\beta }}^{(1)}}(X)\subseteq X \subseteq \overline{B_{V_{5}^{\beta }}^{(1)}}(X)\subseteq \overline{B_{V_{1}^{\beta }}^{(1)}}(X) \subseteq \overline{B_{V_{6}^{\beta }}^{(1)}}(X)$$;
(ii) $$\underline{B_{V_{6}^{\beta }}^{(1)}}(X) \subseteq \underline{B_{V_{2}^{\beta }}^{(1)}}(X)\subseteq \underline{B_{V_{5}^{\beta }}^{(1)}}(X)\subseteq X \subseteq \overline{B_{V_{5}^{\beta }}^{(1)}}(X)\subseteq \overline{B_{V_{2}^{\beta }}^{(1)}}(X) \subseteq \overline{B_{V_{6}^{\beta }}^{(1)}}(X)$$;
(iii) $$\underline{B_{V_{8}^{\beta }}^{(1)}}(X) \subseteq \underline{B_{V_{3}^{\beta }}^{(1)}}(X)\subseteq \underline{B_{V_{7}^{\beta }}^{(1)}}(X)\subseteq X \subseteq \overline{B_{V_{7}^{\beta }}^{(1)}}(X)\subseteq \overline{B_{V_{3}^{\beta }}^{(1)}}(X) \subseteq \overline{B_{V_{8}^{\beta }}^{(1)}}(X)$$; (iv) $$\underline{B_{V_{8}^{\beta }}^{(1)}}(X) \subseteq \underline{B_{V_{4}^{\beta }}^{(1)}}(X)\subseteq \underline{B_{V_{7}^{\beta }}^{(1)}}(X)\subseteq X \subseteq \overline{B_{V_{7}^{\beta }}^{(1)}}(X)\subseteq \overline{B_{V_{4}^{\beta }}^{(1)}}(X) \subseteq \overline{B_{V_{8}^{\beta }}^{(1)}}(X)$$.

#### Proof

The claims follow from Proposition 2 and Definition 15. $\square$

#### Definition 16

Let $(U,B,V_{j}^{\beta })$ be a $V_{j}^{\beta }$-neighborhood space ($j=1,2,\cdots ,8$) and $X\subseteq U$. The type-2 rough set based on $V_{j}^{\beta }$-neighborhoods of *X* is the pair $\langle \underline{B_{V_{j}^{\beta }}^{(2)}}(X),\overline{B_{V_{j}^{\beta }}^{(2)}}(X)\rangle$ composed by the following lower and upper approximations:

$$\begin{aligned} \underline{B_{V_{j}^{\beta }}^{(2)}}(X)=\bigcup \{V_{j}^{\beta }(x)\mid V_{j}^{\beta }(x)\subseteq X\};\overline{B_{V_{j}^{\beta }}^{(2)}}(X)=(\underline{B_{V_{j}^{\beta }}^{(2)}}(X^C))^C. \end{aligned}$$

#### Proposition 6

Let $(U,B,V_{j}^{\beta })$ be a $V_{j}^{\beta }$-neighborhood space $(j=1,2,\cdots ,8)$ and $X,Y\subseteq U$. The type-2 lower and upper approximations based on $V_{j}^{\beta }$-neighborhoods satisfy

(i) $$\underline{B_{V_{j}^{\beta }}^{(2)}}(X)=(\overline{B_{V_{j}^{\beta }}^{(2)}}(X^{C}))^{C}$$; $$\overline{B_{V_{j}^{\beta }}^{(2)}}(X)=(\underline{B_{V_{j}^{\beta }}^{(2)}}(X^{C}))^{C}$$;
(ii) $$\underline{B_{V_{j}^{\beta }}^{(2)}}(U)=U$$; $$\underline{B_{V_{j}^{\beta }}^{(2)}}(\emptyset )=\emptyset$$; $$\overline{B_{V_{j}^{\beta }}^{(2)}}(U)=U$$; $$\overline{B_{V_{j}^{\beta }}^{(2)}}(\emptyset )=\emptyset$$;
(iii) $$\underline{B_{V_{j}^{\beta }}^{(2)}}(X)\subseteq X \subseteq \overline{B_{V_{j}^{\beta }}^{(2)}}(X)$$;
(iv) $$X\subseteq Y\Rightarrow \underline{B_{V_{j}^{\beta }}^{(2)}}(X)\subseteq \underline{B_{V_{j}^{\beta }}^{(2)}}(Y)$$, $$\overline{B_{V_{j}^{\beta }}^{(2)}}(X)\subseteq \overline{B_{V_{j}^{\beta }}^{(2)}}(Y)$$;
(v) $$\underline{B_{V_{j}^{\beta }}^{(2)}}(X\cap Y)\subseteq \underline{B_{V_{j}^{\beta }}^{(2)}}(X)\cap \underline{B_{V_{j}^{\beta }}^{(2)}}(Y)$$; $$\overline{B_{V_{j}^{\beta }}^{(2)}}(X\cup Y)\supseteq \overline{B_{V_{j}^{\beta }}^{(2)}}(X)\cup \overline{B_{V_{j}^{\beta }}^{(2)}}(Y)$$;
(vi) $$\underline{B_{V_{j}^{\beta }}^{(2)}}(X\cup Y)\supseteq \underline{B_{V_{j}^{\beta }}^{(2)}}(X)\cup \underline{B_{V_{j}^{\beta }}^{(2)}}(Y)$$; $$\overline{B_{V_{j}^{\beta }}^{(2)}}(X\cap Y)\subseteq \overline{B_{V_{j}^{\beta }}^{(2)}}(X)\cap \overline{B_{V_{j}^{\beta }}^{(2)}}(Y)$$.
(vii) $$\underline{B_{V_{j}^{\beta }}^{(2)}}(X)= \underline{B_{V_{j}^{\beta }}^{(2)}}(\underline{B_{V_{j}^{\beta }}^{(2)}}(X))$$; $$\overline{B_{V_{j}^{\beta }}^{(2)}}(\overline{B_{V_{j}^{\beta }}^{(2)}}(X))= \overline{B_{V_{j}^{\beta }}^{(2)}}(X)$$.

#### Proof

The proofs of (i)–(iv) and (vi) are similar to Proposition 4.

(v) Let $x\in \underline{B_{V_{j}^{\beta }}^{(2)}}(X\cap Y)$. Then, there exists $V_{j}^{\beta }(y)$, such that $x\in V_{j}^{\beta }(y)\subseteq X \cap Y$. So $V_{j}^{\beta }(y)\subseteq X$ and $V_{j}^{\beta }(y)\subseteq Y$, which induces that $x\in \underline{B_{V_{j}^{\beta }}^{(2)}}(X)$ and $x\in \underline{B_{V_{j}^{\beta }}^{(2)}}(Y)$. Hence, $\underline{B_{V_{j}^{\beta }}^{(2)}}(X\cap Y)\subseteq \underline{B_{V_{j}^{\beta }}^{(2)}}(X)\cap \underline{B_{V_{j}^{\beta }}^{(2)}}(Y)$.

Similarly, let $x\in \underline{B_{V_{j}^{\beta }}^{(2)}}((X \cup Y)^{C})$. Then, there exists $V_{j}^{\beta }(y)$, such that $x\in V_{j}^{\beta }(y)\subseteq (X \cup Y)^{C}=X^{C} \cap Y^{C}$. Therefore, $V_{j}^{\beta }(y)\subseteq X^{C}$ and $V_{j}^{\beta }(y)\subseteq Y^{C}$. Therefore, $x\in \underline{B_{V_{j}^{\beta }}^{(2)}}(X^{C}) \cap \underline{B_{V_{j}^{\beta }}^{(2)}}(Y^{C})$. Therefore, $\underline{B_{V_{j}^{\beta }}^{(2)}}((X \cup Y)^{C}) \subseteq \underline{B_{V_{j}^{\beta }}^{(2)}}(X^{C}) \cap \underline{B_{V_{j}^{\beta }}^{(2)}}(Y^{C})$. That means $(\underline{B_{V_{j}^{\beta }}^{(2)}}((X \cup Y)^{C}))^{C}\supseteq (\underline{B_{V_{j}^{\beta }}^{(2)}}(X^{C}) \cap \underline{B_{V_{j}^{\beta }}^{(2)}}(Y^{C}))^{C}=(\underline{B_{V_{j}^{\beta }}^{(2)}}(X^{C})^{C} \cup (\underline{B_{V_{j}^{\beta }}^{(2)}}(Y^{C}))^{C}$. Hence, $\overline{B_{V_{j}^{\beta }}^{(2)}}(X\cup Y)\supseteq \overline{B_{V_{j}^{\beta }}^{(2)}}(X)\cup \overline{B_{V_{j}^{\beta }}^{(2)}}(Y)$.

(vii) Based on (iii) and (iv), we have $\underline{B_{V_{j}^{\beta }}^{(2)}}(X)\supseteq \underline{B_{V_{j}^{\beta }}^{(2)}}(\underline{B_{V_{j}^{\beta }}^{(2)}}(X))$. Now, we prove $\underline{B_{V_{j}^{\beta }}^{(2)}}(X)\subseteq \underline{B_{V_{j}^{\beta }}^{(2)}}(\underline{B_{V_{j}^{\beta }}^{(2)}}(X))$. Let $x\in \underline{B_{V_{j}^{\beta }}^{(2)}}(X)$. Then, there exists $V_{j}^{\beta }(y)$, such that $x\in V_{j}^{\beta }(y)\subseteq X$. And for any $z\in V_{j}^{\beta }(y)$, $z\in \underline{B_{V_{j}^{\beta }}^{(2)}}(X)$. Therefore, $V_{j}^{\beta }(y)\subseteq \underline{B_{V_{j}^{\beta }}^{(2)}}(X)$. That means $x\in \underline{B_{V_{j}^{\beta }}^{(2)}}(\underline{B_{V_{j}^{\beta }}^{(2)}}(X))$. Hence, $\underline{B_{V_{j}^{\beta }}^{(2)}}(X)\subseteq \underline{B_{V_{j}^{\beta }}^{(2)}}(\underline{B_{V_{j}^{\beta }}^{(2)}}(X))$.

Similarly, $$\overline{B_{V_{j}^{\beta }}^{(2)}}(\overline{B_{V_{j}^{\beta }}^{(2)}}(X))= \overline{B_{V_{j}^{\beta }}^{(2)}}(X)$$. $$\square$$

#### Example 3

For $(U,B,V_{j}^{0.6})$ in Example 1, the type-2 lower and upper approximations based on $V_{j}^{0.6}$-neighborhoods are given in Table 7.

**Table 7:** Type-2 rough approximations based on $V_{j}^{0.6}$-neighborhoods in Example 3

*X*	$\underline{B_{V_{1}^{0.6}}^{(2)}}(X)$	$\overline{B_{V_{1}^{0.6}}^{(2)}}(X)$	$\underline{B_{V_{2}^{0.6}}^{(2)}}(X)$	$\overline{B_{V_{2}^{0.6}}^{(2)}}(X)$	$\underline{B_{V_{3}^{0.6}}^{(2)}}(X)$	$\overline{B_{V_{3}^{0.6}}^{(2)}}(X)$
$\{x_{3}\}$	$\emptyset$	$\{x_{3}\}$	$\emptyset$	$\{x_{3}\}$	$\{x_{3}\}$	*U*
$\{x_{4}\}$	$\emptyset$	$\{x_{4}\}$	$\{x_{4}\}$	$\{x_{3},x_{4}\}$	$\emptyset$	$\{x_{4}\}$
$\{x_{2},x_{3}\}$	$\emptyset$	$\{x_{2},x_{3},x_{4}\}$	$\emptyset$	$\{x_{1},x_{2},x_{3}\}$	$\{x_{3}\}$	*U*
$\{x_{2},x_{5}\}$	$\{x_{5}\}$	*U*	$\{x_{5}\}$	$\{x_{1},x_{2},x_{3},x_{5}\}$	$\emptyset$	$\{x_{1},x_{2},x_{5}\}$
$\{x_{3},x_{4}\}$	$\emptyset$	$\{x_{2},x_{3},x_{4}\}$	$\{x_{4}\}$	$\{x_{3},x_{4}\}$	$\{x_{3},x_{4}\}$	*U*
$\{x_{3},x_{5}\}$	$\{x_{5}\}$	*U*	$\{x_{5}\}$	$\{x_{3},x_{5}\}$	$\{x_{3},x_{5}\}$	*U*
$\{x_{2},x_{3},x_{4}\}$	$\emptyset$	$\{x_{2},x_{3},x_{4}\}$	$\{x_{4}\}$	$\{x_{1},x_{2},x_{3},x_{4}\}$	$\{x_{3},x_{4}\}$	*U*
$\{x_{2},x_{3},x_{5}\}$	$\{x_{2},x_{3},x_{5}\}$	*U*	$\{x_{5}\}$	$\{x_{1},x_{2},x_{3},x_{5}\}$	$\{x_{3},x_{5}\}$	*U*
$\{x_{2},x_{4},x_{5}\}$	$\{x_{2},x_{4},x_{5}\}$	*U*	$\{x_{4},x_{5}\}$	*U*	$\emptyset$	$\{x_{1},x_{2},x_{4},x_{5}\}$

*X*	$\underline{B_{V_{4}^{0.6}}^{(2)}}(X)$	$\overline{B_{V_{4}^{0.6}}^{(2)}}(X)$	$\underline{B_{V_{5}^{0.6}}^{(2)}}(X)$	$\overline{B_{V_{5}^{0.6}}^{(2)}}(X)$	$\underline{B_{V_{6}^{0.6}}^{(2)}}(X)$	$\overline{B_{V_{6}^{0.6}}^{(2)}}(X)$
$\{x_{3}\}$	$\emptyset$	$\{x_{3}\}$	$\emptyset$	$\{x_{3}\}$	$\emptyset$	$\{x_{3}\}$
$\{x_{4}\}$	$\emptyset$	$\{x_{4}\}$	$\{x_{4}\}$	$\{x_{4}\}$	$\emptyset$	$\{x_{3},x_{4}\}$
$\{x_{2},x_{3}\}$	$\emptyset$	$\{x_{2},x_{3},x_{4}\}$	$\{x_{2}\}$	$\{x_{2},x_{3}\}$	$\emptyset$	$\{x_{1},x_{2},x_{3},x_{4}\}$
$\{x_{2},x_{5}\}$	$\{x_{2},x_{5}\}$	*U*	$\{x_{2},x_{5}\}$	$\{x_{2},x_{3},x_{5}\}$	$\{x_{5}\}$	*U*
$\{x_{3},x_{4}\}$	$\emptyset$	$\{x_{3},x_{4}\}$	$\{x_{4}\}$	$\{x_{3},x_{4}\}$	$\emptyset$	$\{x_{3},x_{4}\}$
$\{x_{3},x_{5}\}$	$\{x_{5}\}$	*U*	$\{x_{5}\}$	$\{x_{3},x_{5}\}$	$\{x_{5}\}$	*U*
$\{x_{2},x_{3},x_{4}\}$	$\emptyset$	$\{x_{2},x_{3},x_{4}\}$	$\{x_{2},x_{4}\}$	$\{x_{2},x_{3},x_{4}\}$	$\emptyset$	$\{x_{1},x_{2},x_{3},x_{4}\}$
$\{x_{2},x_{3},x_{5}\}$	$\{x_{2},x_{3},x_{5}\}$	*U*	$\{x_{2},x_{3},x_{5}\}$	$\{x_{2},x_{3},x_{5}\}$	$\{x_{5}\}$	*U*
$\{x_{2},x_{4},x_{5}\}$	$\{x_{2},x_{4},x_{5}\}$	*U*	$\{x_{2},x_{4},x_{5}\}$	$\{x_{2},x_{3},x_{4},x_{5}\}$	$\{x_{2},x_{4},x_{5}\}$	*U*

*X*	$\underline{B_{V_{7}^{0.6}}^{(2)}}(X)$	$\overline{B_{V_{7}^{0.6}}^{(2)}}(X)$	$\underline{B_{V_{8}^{0.6}}^{(2)}}(X)$	$\overline{B_{V_{8}^{0.6}}^{(2)}}(X)$		
$\{x_{3}\}$	$\{x_{3}\}$	$\{x_{3}\}$	$\emptyset$	*U*		
$\{x_{4}\}$	$\{x_{4}\}$	$\{x_{4}\}$	$\emptyset$	$\{x_{4}\}$		
$\{x_{2},x_{3}\}$	$\{x_{2},x_{3}\}$	$\{x_{2},x_{3}\}$	$\emptyset$	*U*		
$\{x_{2},x_{5}\}$	$\{x_{2},x_{5}\}$	$\{x_{2},x_{5}\}$	$\emptyset$	*U*		
$\{x_{3},x_{4}\}$	$\{x_{3},x_{4}\}$	$\{x_{3},x_{4}\}$	$\emptyset$	*U*		
$\{x_{3},x_{5}\}$	$\{x_{3},x_{5}\}$	$\{x_{3},x_{5}\}$	$\{x_{3},x_{5}\}$	*U*		
$\{x_{2},x_{3},x_{4}\}$	$\{x_{2},x_{3},x_{4}\}$	$\{x_{2},x_{3},x_{4}\}$	$\emptyset$	*U*		
$\{x_{2},x_{3},x_{5}\}$	$\{x_{2},x_{3},x_{5}\}$	$\{x_{2},x_{3},x_{5}\}$	$\{x_{2},x_{3},x_{5}\}$	*U*		
$\{x_{2},x_{4},x_{5}\}$	$\{x_{2},x_{4},x_{5}\}$	$\{x_{2},x_{4},x_{5}\}$	$\emptyset$	*U*		

For $X=\{x_{3}\}$ and $Y=\{x_{4}\}$, $X \cup Y=\{x_{3},x_{4}\}$. From Table 7, $\overline{B_{V_{1}^{0.6}}^{(2)}}(X)=\{x_{3}\}$, $\overline{B_{V_{1}^{0.6}}^{(2)}}(Y)=\{x_{4}\}$ and $\overline{B_{V_{1}^{0.6}}^{(2)}}(X \cup Y)=\{x_{2},x_{3},x_{4}\}$. Therefore, $\overline{B_{V_{1}^{0.6}}^{(2)}}(X \cup Y)\not \subseteq \overline{B_{V_{1}^{0.6}}^{(2)}}(X) \cup \overline{B_{V_{1}^{0.6}}^{(2)}}(Y)$. And for $Z=\{x_{2},x_{3},x_{5}\}$ and $T=\{x_{2},x_{4},x_{5}\}$, $Z\cap T=\{x_{2},x_{5}\}$. From Table 7, $\underline{B_{V_{1}^{0.6}}^{(2)}}(Z)=\{x_{2},x_{3},x_{5}\}$, $\underline{B_{V_{1}^{0.6}}^{(2)}}(T)=\{x_{2},x_{4},x_{5}\}$, and $\underline{B_{V_{1}^{0.6}}^{(2)}}(Z \cap T)=\{x_{5}\}$. Therefore, $\underline{B_{V_{1}^{0.6}}^{(2)}}(Z \cap T) \not \supseteq \underline{B_{V_{1}^{0.6}}^{(2)}}(Z) \cap \underline{B_{V_{1}^{0.6}}^{(2)}}(T)$.

#### Proposition 7

Let $(U,B,V_{j}^{\beta })$ be a $V_{j}^{\beta }$-neighborhood space $(j=1,2,\cdots ,8)$ and $X\subseteq U$. The eight type-2 rough sets based on $V_{j}^{\beta }$-neighborhoods satisfy the following properties:

(i) $$\underline{B_{V_{6}^{\beta }}^{(2)}}(X) \subseteq \underline{B_{V_{1}^{\beta }}^{(2)}}(X)\subseteq \underline{B_{V_{5}^{\beta }}^{(2)}}(X)\subseteq X \subseteq \overline{B_{V_{5}^{\beta }}^{(2)}}(X)\subseteq \overline{B_{V_{1}^{\beta }}^{(2)}}(X) \subseteq \overline{B_{V_{6}^{\beta }}^{(2)}}(X)$$;
(ii) $$\underline{B_{V_{6}^{\beta }}^{(2)}}(X) \subseteq \underline{B_{V_{2}^{\beta }}^{(2)}}(X)\subseteq \underline{B_{V_{5}^{\beta }}^{(2)}}(X)\subseteq X \subseteq \overline{B_{V_{5}^{\beta }}^{(2)}}(X)\subseteq \overline{B_{V_{2}^{\beta }}^{(2)}}(X) \subseteq \overline{B_{V_{6}^{\beta }}^{(2)}}(X)$$;
(iii) $$\underline{B_{V_{8}^{\beta }}^{(2)}}(X) \subseteq \underline{B_{V_{3}^{\beta }}^{(2)}}(X)\subseteq \underline{B_{V_{7}^{\beta }}^{(2)}}(X)\subseteq X \subseteq \overline{B_{V_{7}^{\beta }}^{(2)}}(X)\subseteq \overline{B_{V_{3}^{\beta }}^{(2)}}(X) \subseteq \overline{B_{V_{8}^{\beta }}^{(2)}}(X)$$;
(iv) $$\underline{B_{V_{8}^{\beta }}^{(2)}}(X) \subseteq \underline{B_{V_{4}^{\beta }}^{(2)}}(X)\subseteq \underline{B_{V_{7}^{\beta }}^{(2)}}(X)\subseteq X \subseteq \overline{B_{V_{7}^{\beta }}^{(2)}}(X)\subseteq \overline{B_{V_{4}^{\beta }}^{(2)}}(X) \subseteq \overline{B_{V_{8}^{\beta }}^{(2)}}(X)$$.

#### Proof

The claims follow from Proposition 2 and Definition 16 easily. $\square$

#### Definition 17

Let $(U,B,V_{j}^{\beta })$ be a $V_{j}^{\beta }$-neighborhood space ($j=1,2,\cdots ,8$) and $X\subseteq U$. The type-3 rough set based on $V_{j}^{\beta }$-neighborhoods of *X* is the pair $\langle \underline{B_{V_{j}^{\beta }}^{(3)}}(X),\overline{B_{V_{j}^{\beta }}^{(3)}}(X)\rangle$ composed by the following lower and upper approximations:

$$\begin{aligned} \underline{B_{V_{j}^{\beta }}^{(3)}}(X)=(\overline{B_{V_{j}^{\beta }}^{(3)}}(X^C))^C;\overline{B_{V_{j}^{\beta }}^{(3)}}(X)=\bigcup \{V_{j}^{\beta }(x)\mid V_{j}^{\beta }(x)\cap X \ne \emptyset \}. \end{aligned}$$

#### Proposition 8

Let $(U,B,V_{j}^{\beta })$ be a $V_{j}^{\beta }$-neighborhood space $(j=1,2,\cdots ,8)$ and $X,Y\subseteq U$. The type-3 lower and upper approximations based on $V_{j}^{\beta }$-neighborhoods satisfy the following:

(i) $$\underline{B_{V_{j}^{\beta }}^{(3)}}(X)=(\overline{B_{V_{j}^{\beta }}^{(3)}}(X^{C}))^{C}$$; $$\overline{B_{V_{j}^{\beta }}^{(3)}}(X)=(\underline{B_{V_{j}^{\beta }}^{(3)}}(X^{C}))^{C}$$;
(ii) $$\underline{B_{V_{j}^{\beta }}^{(3)}}(U)=U$$; $$\underline{B_{V_{j}^{\beta }}^{(3)}}(\emptyset )=\emptyset$$; $$\overline{B_{V_{j}^{\beta }}^{(3)}}(U)=U$$; $$\overline{B_{V_{j}^{\beta }}^{(3)}}(\emptyset )=\emptyset$$;
(iii) $$\underline{B_{V_{j}^{\beta }}^{(3)}}(X)\subseteq X \subseteq \overline{B_{V_{j}^{\beta }}^{(3)}}(X)$$;
(iv) $$X\subseteq Y\Rightarrow \underline{B_{V_{j}^{\beta }}^{(3)}}(X)\subseteq \underline{B_{V_{j}^{\beta }}^{(3)}}(Y)$$, $$\overline{B_{V_{j}^{\beta }}^{(3)}}(X)\subseteq \overline{B_{V_{j}^{\beta }}^{(3)}}(Y)$$;
(v) $$\underline{B_{V_{j}^{\beta }}^{(3)}}(X\cap Y)=\underline{B_{V_{j}^{\beta }}^{(3)}}(X)\cap \underline{B_{V_{j}^{\beta }}^{(3)}}(Y)$$; it $$\overline{B_{V_{j}^{\beta }}^{(3)}}(X\cup Y)=\overline{B_{V_{j}^{\beta }}^{(3)}}(X)\cup \overline{B_{V_{j}^{\beta }}^{(3)}}(Y)$$;
(vi) $$\underline{B_{V_{j}^{\beta }}^{(3)}}(X\cup Y)\supseteq \underline{B_{V_{j}^{\beta }}^{(3)}}(X)\cup \underline{B_{V_{j}^{\beta }}^{(3)}}(Y)$$; $$\overline{B_{V_{j}^{\beta }}^{(3)}}(X\cap Y)\subseteq \overline{B_{V_{j}^{\beta }}^{(3)}}(X)\cap \overline{B_{V_{j}^{\beta }}^{(3)}}(Y)$$;
(vii) $$\underline{B_{V_{j}^{\beta }}^{(3)}}(\underline{B_{V_{j}^{\beta }}^{(3)}}(X))\subseteq \underline{B_{V_{j}^{\beta }}^{(3)}}(X)$$; $$\overline{B_{V_{j}^{\beta }}^{(3)}}(X)\subseteq \overline{B_{V_{j}^{\beta }}^{(3)}}(\overline{B_{V_{j}^{\beta }}^{(3)}}(X))$$;
(viii) $$X\subseteq \underline{B_{V_{j}^{\beta }}^{(3)}}(\overline{B_{V_{j}^{\beta }}^{(3)}}(X))$$; $$\overline{B_{V_{j}^{\beta }}^{(3)}}(\underline{B_{V_{j}^{\beta }}^{(3)}}(X))\subseteq X$$.

#### Proof

Similar to Proposition 4 and 6. $\square$

#### Example 4

For $(U,B,V_{j}^{0.6})$ in Example 1, the type-3 lower and upper approximations based on $V_{j}^{0.6}$-neighborhoods are given in Table 8.

**Table 8:** Type-3 rough approximations based on $V_{j}^{0.6}$-neighborhoods in Example 4

*X*	$\underline{B_{V_{1}^{0.6}}^{(3)}}(X)$	$\overline{B_{V_{1}^{0.6}}^{(3)}}(X)$	$\underline{B_{V_{2}^{0.6}}^{(3)}}(X)$	$\overline{B_{V_{2}^{0.6}}^{(3)}}(X)$	$\underline{B_{V_{3}^{0.6}}^{(3)}}(X)$	$\overline{B_{V_{3}^{0.6}}^{(3)}}(X)$
$\{x_{3}\}$	$\emptyset$	$\{x_{2},x_{3},x_{4},x_{5}\}$	$\emptyset$	*U*	$\{x_{3}\}$	*U*
$\{x_{4}\}$	$\emptyset$	$\{x_{2},x_{3},x_{4},x_{5}\}$	$\emptyset$	*U*	$\emptyset$	$\{x_{3},x_{4}\}$
$\{x_{2},x_{3}\}$	$\emptyset$	$\{x_{2},x_{3},x_{4},x_{5}\}$	$\emptyset$	*U*	$\emptyset$	*U*
$\{x_{2},x_{5}\}$	$\emptyset$	*U*	$\emptyset$	*U*	$\emptyset$	$\{x_{1},x_{2},x_{3},x_{5}\}$
$\{x_{3},x_{4}\}$	$\emptyset$	$\{x_{2},x_{3},x_{4},x_{5}\}$	$\emptyset$	*U*	$\{x_{4}\}$	*U*
$\{x_{3},x_{5}\}$	$\emptyset$	*U*	$\emptyset$	*U*	$\{x_{5}\}$	*U*
$\{x_{2},x_{3},x_{4}\}$	$\emptyset$	$\{x_{2},x_{3},x_{4},x_{5}\}$	$\emptyset$	*U*	$\{x_{4}\}$	*U*
$\{x_{2},x_{3},x_{5}\}$	$\emptyset$	*U*	$\emptyset$	*U*	$\{x_{5}\}$	*U*
$\{x_{2},x_{4},x_{5}\}$	$\emptyset$	*U*	$\emptyset$	*U*	$\emptyset$	*U*

*X*	$\underline{B_{V_{4}^{0.6}}^{(3)}}(X)$	$\overline{B_{V_{4}^{0.6}}^{(3)}}(X)$	$\underline{B_{V_{5}^{0.6}}^{(3)}}(X)$	$\overline{B_{V_{5}^{0.6}}^{(3)}}(X)$	$\underline{B_{V_{6}^{0.6}}^{(3)}}(X)$	$\overline{B_{V_{6}^{0.6}}^{(3)}}(X)$
$\{x_{3}\}$	$\emptyset$	$\{x_{2},x_{3},x_{5}\}$	$\emptyset$	$\{x_{2},x_{3},x_{5}\}$	$\emptyset$	*U*
$\{x_{4}\}$	$\emptyset$	$\{x_{2},x_{4},x_{5}\}$	$\{x_{4}\}$	$\{x_{4}\}$	$\emptyset$	*U*
$\{x_{2},x_{3}\}$	$\emptyset$	$\{x_{2},x_{3},x_{4},x_{5}\}$	$\emptyset$	$\{x_{2},x_{3},x_{5}\}$	$\emptyset$	*U*
$\{x_{2},x_{5}\}$	$\emptyset$	*U*	$\emptyset$	$\{x_{2},x_{3},x_{5}\}$	$\emptyset$	*U*
$\{x_{3},x_{4}\}$	$\emptyset$	$\{x_{2},x_{3},x_{4},x_{5}\}$	$\{x_{4}\}$	$\{x_{2},x_{3},x_{4},x_{5}\}$	$\emptyset$	*U*
$\{x_{3},x_{5}\}$	$\emptyset$	*U*	$\emptyset$	$\{x_{2},x_{3},x_{5}\}$	$\emptyset$	*U*
$\{x_{2},x_{3},x_{4}\}$	$\emptyset$	$\{x_{2},x_{3},x_{4},x_{5}\}$	$\{x_{4}\}$	$\{x_{2},x_{3},x_{4},x_{5}\}$	$\emptyset$	*U*
$\{x_{2},x_{3},x_{5}\}$	$\{x_{3}\}$	*U*	$\{x_{2},x_{3},x_{5}\}$	$\{x_{2},x_{3},x_{5}\}$	$\emptyset$	*U*
$\{x_{2},x_{4},x_{5}\}$	$\{x_{4}\}$	$\{x_{2},x_{3},x_{4},x_{5}\}$	$\{x_{4}\}$	$\{x_{2},x_{3},x_{4},x_{5}\}$	$\emptyset$	*U*

*X*	$\underline{B_{V_{7}^{0.6}}^{(3)}}(X)$	$\overline{B_{V_{7}^{0.6}}^{(3)}}(X)$	$\underline{B_{V_{8}^{0.6}}^{(3)}}(X)$	$\overline{B_{V_{8}^{0.6}}^{(3)}}(X)$		
$\{x_{3}\}$	$\{x_{3}\}$	$\{x_{3}\}$	$\emptyset$	*U*		
$\{x_{4}\}$	$\{x_{4}\}$	$\{x_{4}\}$	$\emptyset$	$\{x_{2},x_{3},x_{4},x_{5}\}$		
$\{x_{2},x_{3}\}$	$\{x_{2},x_{3}\}$	$\{x_{2},x_{3}\}$	$\emptyset$	*U*		
$\{x_{2},x_{5}\}$	$\{x_{2},x_{5}\}$	$\{x_{2},x_{5}\}$	$\emptyset$	*U*		
$\{x_{3},x_{4}\}$	$\{x_{3},x_{4}\}$	$\{x_{3},x_{4}\}$	$\emptyset$	*U*		
$\{x_{3},x_{5}\}$	$\{x_{3},x_{5}\}$	$\{x_{3},x_{5}\}$	$\emptyset$	*U*		
$\{x_{2},x_{3},x_{4}\}$	$\{x_{2},x_{3},x_{4}\}$	$\{x_{2},x_{3},x_{4}\}$	$\emptyset$	*U*		
$\{x_{2},x_{3},x_{5}\}$	$\{x_{2},x_{3},x_{5}\}$	$\{x_{2},x_{3},x_{5}\}$	$\emptyset$	*U*		
$\{x_{2},x_{4},x_{5}\}$	$\{x_{2},x_{4},x_{5}\}$	$\{x_{2},x_{4},x_{5}\}$	$\emptyset$	*U*		

For $Y=\{x_{4}\}$, Table 8 shows $\overline{B_{V_{3}^{0.6}}^{(3)}}(Y)=\{x_{3},x_{4}\}$, $\overline{B_{V_{3}^{0.6}}^{(3)}}(\{x_{3},x_{4}\})=U$. Therefore, $\overline{B_{V_{3}^{0.6}}^{(3)}}(\overline{B_{V_{3}^{0.6}}^{(3)}}(Y))\supseteq \overline{B_{V_{3}^{0.6}}^{(3)}}(Y)$. And for $Z=\{x_{2},x_{3},x_{5}\}$, $\underline{B_{V_{4}^{0.6}}^{(3)}}(Z)=\{x_{3}\}$, $\underline{B_{V_{4}^{0.6}}^{(3)}}(\{x_{3}\})=\emptyset$. Therefore, $\underline{B_{V_{4}^{0.6}}^{(3)}}(\underline{B_{V_{4}^{0.6}}^{(3)}}(Z))\subseteq \underline{B_{V_{4}^{0.6}}^{(3)}}(Z)$.

#### Proposition 9

Let $(U,B,V_{j}^{\beta })$ be a $V_{j}^{\beta }$-neighborhood space $(j=1,2,\cdots ,8)$ and $X\subseteq U$. The eight type-3 rough sets based on $V_{j}^{\beta }$-neighborhoods satisfy the following properties:

(i) $$\underline{B_{V_{6}^{\beta }}^{(3)}}(X) \subseteq \underline{B_{V_{1}^{\beta }}^{(3)}}(X)\subseteq \underline{B_{V_{5}^{\beta }}^{(3)}}(X)\subseteq X \subseteq \overline{B_{V_{5}^{\beta }}^{(3)}}(X)\subseteq \overline{B_{V_{1}^{\beta }}^{(3)}}(X) \subseteq \overline{B_{V_{6}^{\beta }}^{(3)}}(X)$$;
(ii) $$\underline{B_{V_{6}^{\beta }}^{(3)}}(X) \subseteq \underline{B_{V_{2}^{\beta }}^{(3)}}(X)\subseteq \underline{B_{V_{5}^{\beta }}^{(3)}}(X)\subseteq X \subseteq \overline{B_{V_{5}^{\beta }}^{(3)}}(X)\subseteq \overline{B_{V_{2}^{\beta }}^{(3)}}(X) \subseteq \overline{B_{V_{6}^{\beta }}^{(3)}}(X)$$;
(iii) $$\underline{B_{V_{8}^{\beta }}^{(3)}}(X) \subseteq \underline{B_{V_{3}^{\beta }}^{(3)}}(X)\subseteq \underline{B_{V_{7}^{\beta }}^{(3)}}(X)\subseteq X \subseteq \overline{B_{V_{7}^{\beta }}^{(3)}}(X)\subseteq \overline{B_{V_{3}^{\beta }}^{(3)}}(X) \subseteq \overline{B_{V_{8}^{\beta }}^{(3)}}(X)$$;
(iv) $$\underline{B_{V_{8}^{\beta }}^{(3)}}(X) \subseteq \underline{B_{V_{4}^{\beta }}^{(3)}}(X)\subseteq \underline{B_{V_{7}^{\beta }}^{(3)}}(X)\subseteq X \subseteq \overline{B_{V_{7}^{\beta }}^{(3)}}(X)\subseteq \overline{B_{V_{4}^{\beta }}^{(3)}}(X) \subseteq \overline{B_{V_{8}^{\beta }}^{(3)}}(X)$$.

#### Proof

The conclusions follow from Proposition 2 and Definition 17. $\square$

#### Proposition 10

Let $(U,B,V_{j}^{\beta })$ be a $V_{j}^{\beta }$-neighborhood space $(j=1,2,\cdots ,8)$ and $X\subseteq U$. Then, for any $j=1,\cdots ,8$

$$\begin{aligned} \underline{B_{V_{j}^{\beta }}^{(3)}}(X)\subseteq \underline{B_{V_{j}^{\beta }}^{(1)}}(X)\subseteq \underline{B_{V_{j}^{\beta }}^{(2)}}(X)\subseteq X\subseteq \overline{B_{V_{j}^{\beta }}^{(2)}}(X)\subseteq \overline{B_{V_{j}^{\beta }}^{(1)}}(X)\subseteq \overline{B_{V_{j}^{\beta }}^{(3)}}(X). \end{aligned}$$

#### Proof

Since $V_{j}^{\beta }$-neighborhood operator is reflexive, the claim is obvious by Theorem 7 in  Yao (1998). $\square$

## Topology structure of $V_{j}^{\beta }$-neighborhood space

 Kelley (1995) proposed that if ${\mathscr {S}}$ is any non-void family of sets, the family of all finite intersections of members of ${\mathscr {S}}$ is the base for a topology for the set $U=\bigcup \{S\mid S\in {\mathscr {S}}\}$. Based on it, we present the following topology structure analysis of $V_{j}^{\beta }$-neighborhood space.

### Proposition 11

Let $(U,B,V_{j}^{\beta })$ be a $V_{j}^{\beta }$-neighborhood space $(j=1,2,\cdots ,8)$. Define ${\mathscr {U}}_{j}(x)=\{X \subseteq U \vert V_{j}^{\beta }(x)\subseteq X\}$ and ${\mathscr {T}}_{j}=\{{\mathscr {U}}_{j}(x)\vert x\in U\}$. Then,

(i) ${\mathscr {T}}_{j}$ is a topology of *U*, and $\{V_{j}^{\beta }(x)\}$ is a base of ${\mathscr {U}}_{j}(x)$;

(ii) $${\mathscr {T}}_{6}\subseteq {\mathscr {T}}_{1}\subseteq {\mathscr {T}}_{5}$$; $${\mathscr {T}}_{6}\subseteq {\mathscr {T}}_{2}\subseteq {\mathscr {T}}_{5}$$; $${\mathscr {T}}_{8}\subseteq {\mathscr {T}}_{3}\subseteq {\mathscr {T}}_{7}$$; $${\mathscr {T}}_{8}\subseteq {\mathscr {T}}_{4}\subseteq {\mathscr {T}}_{7}$$.

### Proof

(i) For any $j=1,2,\cdots ,8$, obviously, ${\mathscr {T}}_{j}$ satisfies (i), (ii) and (iii) in Definition 11. Therefore, ${\mathscr {T}}_{j}$ is a topology.

Furthermore, based on the definition of ${\mathscr {U}}_{j}(x)$ and Definition 12, we have $\{V_{j}^{\beta }(x)\}$ is a base of ${\mathscr {U}}_{j}(x)$.

(ii) It can be proved by the claim (ii) of Proposition

2. $$\square$$

Pawlak’s rough set is built on topology theory. Let $R$ be an equivalence relation on *U* and $X\subseteq U$. Then, $(U, DIS(R))$ is a topology space, where *DIS*(*R*) is the family of all open and closed sets, and $U/R$ is a base for the topology $DIS(R)$. And the *R*-lower and the *R*-upper approximation of *X* is the interior and closure of *X* in $(U,DIS(R))$, respectively. However, from Propositions 4$\sim$6, we can find in neighborhood space $(U,B,V_{j}^{\beta })$ the three pairs of approximation operators in $V_{j}^{\beta }$-neighborhood spaces cannot totally satisfy Kuratowski axiom (Theorem 1). In the following, we discuss the conditions under which $\underline{B_{V_{j}^{\beta }}^{(k)}}$ and $\overline{B_{V_{j}^{\beta }}^{(k)}}$ ($k=1,2,3$ and $j=1,2,\cdots ,8$) are interior and closure operators, respectively.

### Proposition 12

Let $(U,B,V_{j}^{\beta })$ be a $V_{j}^{\beta }$-neighborhood space $(j=1,2,\cdots ,8)$. If $V_{j}^{\beta }$-neighborhood is transitive, then $\underline{B_{V_{j}^{\beta }}^{(1)}}$ and $\overline{B_{V_{j}^{\beta }}^{(1)}}$ are interior and closure operators, respectively in $(U,{\mathscr {T}}_{j})$.

### Proof

Based on (vii) of Proposition 4, we only prove that $\underline{B_{V_{j}^{\beta }}^{(1)}}(X)\subseteq \underline{B_{V_{j}^{\beta }}^{(1)}}(\underline{B_{V_{j}^{\beta }}^{(1)}}(X))$ and $\overline{B_{V_{j}^{\beta }}^{(1)}}(\overline{B_{V_{j}^{\beta }}^{(1)}}(X))\subseteq \overline{B_{V_{j}^{\beta }}^{(1)}}(X)$, for any $X\subseteq U$.

For any $x\in \underline{B_{V_{j}^{\beta }}^{(1)}}(X)$, $V_{j}^{\beta }(x)\subseteq X$. If $V_{j}^{\beta }$-neighborhood is transitive, for any $y\in V_{j}^{\beta }(x)$, we have $V_{j}^{\beta }(y)\subseteq V_{j}^{\beta }(x)\subseteq X$. Therefore, $y\in \underline{B_{V_{j}^{\beta }}^{(1)}}(X)$. Then, $V_{j}^{\beta }(y)\subseteq \underline{B_{V_{j}^{\beta }}^{(1)}}(X)$. Therefore, $y\in \underline{B_{V_{j}^{\beta }}^{(1)}}(\underline{B_{V_{j}^{\beta }}^{(1)}}(X))$. Hence, $\underline{B_{V_{j}^{\beta }}^{(1)}}(X)\subseteq \underline{B_{V_{j}^{\beta }}^{(1)}}(\underline{B_{V_{j}^{\beta }}^{(1)}}(X))$.

On the other hand, if $x\in \overline{B_{V_{j}^{\beta }}^{(1)}}(\overline{B_{V_{j}^{\beta }}^{(1)}}(X))$, then $V_{j}^{\beta }(x)\cap \overline{B_{V_{j}^{\beta }}^{(1)}}(X)\ne \emptyset$. Let $y\in V_{j}^{\beta }(x)\cap \overline{B_{V_{j}^{\beta }}^{(1)}}(X)$. Since $V_{j}^{\beta }$-neighborhood is transitive, $V_{j}^{\beta }(y)\subseteq V_{j}^{\beta }(x)$ and $V_{j}^{\beta }(y)\cap X\ne \emptyset$. Therefore, $V_{j}^{\beta }(x)\cap X\ne \emptyset$. Then, $x\in \overline{B_{V_{j}^{\beta }}^{(1)}}(X)$. Hence, $\overline{B_{V_{j}^{\beta }}^{(1)}}(\overline{B_{V_{j}^{\beta }}^{(1)}}(X))\subseteq \overline{B_{V_{j}^{\beta }}^{(1)}}(X)$.

Therefore, $$\underline{B_{V_{j}^{\beta }}^{(1)}}(X)=\underline{B_{V_{j}^{\beta }}^{(1)}}(\underline{B_{V_{j}^{\beta }}^{(1)}}(X))$$ and $$\overline{B_{V_{j}^{\beta }}^{(1)}}(\overline{B_{V_{j}^{\beta }}^{(1)}}(X))=\overline{B_{V_{j}^{\beta }}^{(1)}}(X)$$.

Therefore, $\underline{B_{V_{j}^{\beta }}^{(1)}}$ and $\overline{B_{V_{j}^{\beta }}^{(1)}}$ satisfy Theorem 1 according to Proposition 4, which are interior and closure operators, respectively. $\square$

### Proposition 13

Let $(U,B,V_{j}^{\beta })$ be a $V_{j}^{\beta }$-neighborhood space $(j=1,2,\cdots ,8)$. If $V_{j}^{\beta }$-neighborhood is transitive, then $\underline{B_{V_{j}^{\beta }}^{(2)}}$ and $\overline{B_{V_{j}^{\beta }}^{(2)}}$ are interior and closure operators, respectively, in $(U,{\mathscr {T}}_{j})$.

### Proof

Based on Proposition 6, we only prove $\underline{B_{V_{j}^{\beta }}^{(2)}}(X)\cap \underline{B_{V_{j}^{\beta }}^{(2)}}(Y)\subseteq \underline{B_{V_{j}^{\beta }}^{(2)}}(X \cap Y)$ and $\overline{B_{V_{j}^{\beta }}^{(2)}}(X \cup Y)\subseteq \overline{B_{V_{j}^{\beta }}^{(2)}}(X) \cup \overline{B_{V_{j}^{\beta }}^{(2)}}(Y)$, for any $X\subseteq U$.

For any $x\in \underline{B_{V_{j}^{\beta }}^{(2)}}(X)\cap \underline{B_{V_{j}^{\beta }}^{(2)}}(Y)$, there exist $y,z\in U$, such that $x\in V_{j}^{\beta }(y)\subseteq X$ and $x\in V_{j}^{\beta }(z)\subseteq Y$. Since $V_{j}^{\beta }$-neighborhood is transitive, $V_{j}^{\beta }(x)\subseteq V_{j}^{\beta }(y)$ and $V_{j}^{\beta }(x)\subseteq V_{j}^{\beta }(z)$. Therefore, $x\in V_{j}^{\beta }(x)\subseteq X \cap Y$. That means $\underline{B_{V_{j}^{\beta }}^{(2)}}(X)\cap \underline{B_{V_{j}^{\beta }}^{(2)}}(Y)\subseteq \underline{B_{V_{j}^{\beta }}^{(2)}}(X \cap Y)$. Combined with Claim (iv) in Proposition 6, we have $\underline{B_{V_{j}^{\beta }}^{(2)}}(X)\cap \underline{B_{V_{j}^{\beta }}^{(2)}}(Y)=\underline{B_{V_{j}^{\beta }}^{(2)}}(X \cap Y)$. Similarly, $\overline{B_{V_{j}^{\beta }}^{(2)}}(X)\cup \overline{B_{V_{j}^{\beta }}^{(2)}}(Y)= \overline{B_{V_{j}^{\beta }}^{(2)}}(X \cup Y)$.

Then, we have $\underline{B_{V_{j}^{\beta }}^{(2)}}$ and $\overline{B_{V_{j}^{\beta }}^{(2)}}$ also satisfy Theorem 1 based on Proposition 6, which are interior and closure operators, respectively. $\square$

### Proposition 14

Let $(U,B,V_{j}^{\beta })$ be a $V_{j}^{\beta }$-neighborhood space $(j=1,2,\cdots ,8)$. If $V_{j}^{\beta }$-neighborhood is Euclidean, then $\underline{B_{V_{j}^{\beta }}^{(3)}}$ and $\overline{B_{V_{j}^{\beta }}^{(3)}}$ are interior and closure operators, respectively.

### Proof

Based on Proposition 8, we only prove that $\underline{B_{V_{j}^{\beta }}^{(3)}}(X)\subseteq \underline{B_{V_{j}^{\beta }}^{(3)}}(\underline{B_{V_{j}^{\beta }}^{(3)}}(X))$ and $\overline{B_{V_{j}^{\beta }}^{(3)}}(\overline{B_{V_{j}^{\beta }}^{(3)}}(X))\subseteq \overline{B_{V_{j}^{\beta }}^{(3)}}(X)$, for any $X\subseteq U$.

For any $x\in \overline{B_{V_{j}^{\beta }}^{(3)}}(\overline{B_{V_{j}^{\beta }}^{(3)}}(X))$, there exist $y\in U$, such that $x\in V_{j}^{\beta }(y)$ and $V_{j}^{\beta }(y)\cap \overline{B_{V_{j}^{\beta }}^{(3)}}(X)\ne \emptyset$. Since $V_{j}^{\beta }$-neighborhood is Euclidean, $V_{j}^{\beta }(y)\subseteq V_{j}^{\beta }(x)$, so $V_{j}^{\beta }(x)\cap \overline{B_{V_{j}^{\beta }}^{(3)}}(X)\ne \emptyset$. Let $z\in V_{j}^{\beta }(x)\cap \overline{B_{V_{j}^{\beta }}^{(3)}}(X)$. Then, $z\in V_{j}^{\beta }(x)$ and $z\in \overline{B_{V_{j}^{\beta }}^{(3)}}(X)$. Therefore, $V_{j}^{\beta }(x)\subseteq V_{j}^{\beta }(z)$ and there is $t\in U$, such that $z\in V_{j}^{\beta }(t)$ and $V_{j}^{\beta }(t)\cap X\ne \emptyset$. Since $V_{j}^{\beta }(t)\subseteq V_{j}^{\beta }(z)$, $V_{j}^{\beta }(z)\cap X\ne \emptyset$. Since, for any $s\in V_{j}^{\beta }(z)$, we have $V_{j}^{\beta }(z)\cap X\ne \emptyset$. So $V_{j}^{\beta }(z)\subseteq \overline{B_{V_{j}^{\beta }}^{(3)}}(X)$. And since $x\in V_{j}^{\beta }(x)\subseteq V_{j}^{\beta }(z)$, $x\in \overline{B_{V_{j}^{\beta }}^{(3)}}(X)$. That means $\overline{B_{V_{j}^{\beta }}^{(3)}}(\overline{B_{V_{j}^{\beta }}^{(3)}}(X))\subseteq \overline{B_{V_{j}^{\beta }}^{(3)}}(X)$.

Furthermore, based on (i) of Proposition 8, we have $\underline{B_{V_{j}^{\beta }}^{(3)}}(X)\subseteq \underline{B_{V_{j}^{\beta }}^{(3)}}(\underline{B_{V_{j}^{\beta }}^{(3)}}(X))$.

Therefore, $\underline{B_{V_{j}^{\beta }}^{(3)}}$ and $\overline{B_{V_{j}^{\beta }}^{(3)}}$ also satisfy Theorem 1 based on Proposition 8, which are interior and closure operators, respectively. $\square$

### Proposition 15

Let $(U,B,V_{j}^{\beta })$ $(j=1,2,\cdots ,8)$ be a $V_{j}^{\beta }$-neighborhood space and $X\subseteq U$. If $V_{j}^{\beta }$-neighborhood is transitive, then $\langle \underline{B_{V_{j}^{\beta }}^{(1)}}(X),\overline{B_{V_{j}^{\beta }}^{(1)}}(X)\rangle$ and $\langle \underline{B_{V_{j}^{\beta }}^{(2)}}(X),\overline{B_{V_{j}^{\beta }}^{(2)}}(X)\rangle$ are equivalent; furthermore, if $V_{j}^{\beta }$-neighborhood is both transitive and symmetric, then the three pairs of lower and upper approximation operators are equivalent.

### Proof

(i) First, when $V_{j}^{\beta }$-neighborhood is transitive, we prove $\underline{B_{V_{j}^{\beta }}^{(1)}}(X)=\underline{B_{V_{j}^{\beta }}^{(2)}}(X)$ and $\overline{B_{V_{j}^{\beta }}^{(1)}}(X)=\overline{B_{V_{j}^{\beta }}^{(2)}}(X)$. Based on Proposition 10, we only prove $\underline{B_{V_{j}^{\beta }}^{(2)}}(X)\subseteq \underline{B_{V_{j}^{\beta }}^{(1)}}(X)$ and $\overline{B_{V_{j}^{\beta }}^{(1)}}(X)\subseteq \overline{B_{V_{j}^{\beta }}^{(2)}}(X)$.

For any $x\in \underline{B_{V_{j}^{\beta }}^{(2)}}(X)$, there exists $y\in U$, such that $x\in V_{j}^{\beta }(y)\subseteq X$. Since $V_{j}^{\beta }$ is transitive, $V_{j}^{\beta }(x)\subseteq V_{j}^{\beta }(y)\subseteq X$. Therefore, $x\in \underline{B_{V_{j}^{\beta }}^{(1)}}(X)$. That means $\underline{B_{V_{j}^{\beta }}^{(2)}}(X)\subseteq \underline{B_{V_{j}^{\beta }}^{(1)}}(X)$.

For any $x\in \overline{B_{V_{j}^{\beta }}^{(1)}}(X)$, $V_{j}^{\beta }(x)\cap X\ne \emptyset$. Assume $x\not \in \overline{B_{V_{j}^{\beta }}^{(2)}}(X)$. Then, $x\in \underline{B_{V_{j}^{\beta }}^{(2)}}(X^{C})$. Therefore, there exists $y\in U$, such that $x\in V_{j}^{\beta }(y)\subseteq X^{C}$. Since $V_{j}^{\beta }$ is transitive, $V_{j}^{\beta }(x)\subseteq V_{j}^{\beta }(y)\subseteq X^{C}$, which is contradiction with $V_{j}^{\beta }(x)\cap X\ne \emptyset$. Therefore, $x\in \overline{B_{V_{j}^{\beta }}^{(2)}}(X)$. That means $\overline{B_{V_{j}^{\beta }}^{(1)}}(X)\subseteq \overline{B_{V_{j}^{\beta }}^{(2)}}(X)$.

Hence, $$\underline{B_{V_{j}^{\beta }}^{(1)}}(X)=\underline{B_{V_{j}^{\beta }}^{(2)}}(X)$$ and $$\overline{B_{V_{j}^{\beta }}^{(1)}}(X)=\overline{B_{V_{j}^{\beta }}^{(2)}}(X)$$.

(ii) Since $V_{j}^{\beta }$-neighborhood is reflexive when it is symmetric and transitive, $(U, B, V_{j}^{\beta })$ is an approximation space with the equivalent relation. Similar to the discussion of Corollary 11 in  Yao (1998), the three pairs of lower and upper approximation operators are equivalent. $\square$

## Attribute reduction of rough sets in $V_{j}^{\beta }$-neighborhood spaces

The section aims to show the applicability and flexibility of the proposed rough set models based on variable containment neighborhoods. In classical rough set theory, the boundary regions lead to the inaccuracy of a set. The larger the boundary regions, the weaker the accuracy of the approximation. This paper uses the accuracy measure and dependence measure to depict the applications of the novel rough set models. Considering the relationship between the above three novel rough set models, we only take type-1 rough sets based on $V_{j}^{\beta }$-neighborhoods as the research objects hereinafter.

In the following experiment, all algorithms are run on a personal computer with Windows 10 and Core(TM)i7-10510U CPU 1.8GHz, and 16GB RAM. The software being used is Matlab R2019a.

### Accuracy measures

Accuracy measure is one of the important indicators to characterize the inaccuracy and incompleteness of rough sets.  Allam et al. (2005),  Al-shami (2021) and Al-shami et al. (2021) defined the $N_{j}$-accuracy measure, $E_{j}$-accuracy measure, and $C_{j}$-accuracy measure, which are $\rho _{N_{j}}(X)=\frac{\mid X\cap \underline{B_{N_{j}}}(X)\mid }{\mid X\cup \overline{B_{N_{j}}}(X)\mid }$, $\rho _{E_{j}}(X)=\frac{\mid X\cap \underline{B_{E_{j}}}(X)\mid }{\mid X\cup \overline{B_{E_{j}}}(X)\mid }$ and $\rho _{C_{j}}(X)=\frac{\mid \underline{B_{C_{j}}}(X)\mid }{\mid \overline{B_{C_{j}}}(X)\mid }$, respectively. Combining the characteristics of $V_{j}^{\beta }$-neighborhood-based rough sets, we give the following definition.

#### Definition 18

Let $(U,B,V_{j}^{\beta })$ be a $V_{j}^{\beta }$-neighborhood space and *X* be a nonempty subset of *U*

$$\begin{aligned} \rho _{V_{j}^{\beta }}(X)=\frac{\mid \underline{B_{V_{j}^{\beta }}^{(1)}}(X)\mid }{\mid \overline{B_{V_{j}^{\beta }}^{(1)}}(X)\mid } \end{aligned}$$

is called the $V_{j}^{\beta }$-neighborhood accuracy measure of *X*, where $j=1,2,\cdots ,8$.

#### Proposition 16

Let *B* be a reflexive binary relation on *U*, $X\subseteq U$ and $0<\beta _{1}<\beta _{2}\le 1$. Then, we have

$$\begin{aligned} \rho _{E_{j}}(X)\le \rho _{V_{j}^{\beta _{1}}}(X)\le \rho _{V_{j}^{\beta _{2}}}(X)\le \rho _{C_{j}}(X). \end{aligned}$$

#### Proof

By (iii) of Proposition 3 and Definition 8, 10 and 15, it is true that $\underline{B_{E_{j}}}(X)\subseteq \underline{B_{V_{j}^{\beta _{2}}}^{(1)}}(X)\subseteq \underline{B_{V_{j}^{\beta _{2}}}^{(1)}}(X)\subseteq \underline{B_{C_{j}}}(X)\subseteq X \subseteq \overline{B_{C_{j}}}(X) \subseteq \overline{B_{V_{j}^{\beta _{2}}}^{(1)}}(X) \subseteq \overline{B_{V_{j}^{\beta _{1}}}^{(1)}}(X) \subseteq \overline{B_{E_{j}}}(X)$. Therefore, the conclusion holds. $\square$

The following example proves the superiority of the novel accuracy measure compared with the existing approaches in Al-shami (2021) and Al-shami et al. (2021).

#### Example 5

We use the data in Example 5.6 of  Al-shami (2021), which gave the five symptoms of heart diseases for 12 patients and constructed the $N_{1}$-neighborhood, $C_{1}$-neighborhood, and $E_{1}$-neighborhood of each patient. We continue to calculate the $V_{1}^{0.75}$, $V_{1}^{0.8}$-neighborhoods in Table 9.

**Table 9:** $N_{1}$, $C_{1}$, $V_{1}^{0.8}$, $V_{1}^{0.75}$, and $E_{1}$-neighborhoods in Example 5

*X*	$N_{1}$	$C_{1}$	$V_{1}^{0.8}$	$V_{1}^{0.75}$	$E_{1}$
$\{x_{1}\}$	$\{x_{1},x_{3},x_{7},x_{9},x_{11}\}$	$\{x_{1}\}$	$\{x_{1}\}$	$\{x_{1},x_{3},x_{7}\}$	$\{x_{1},x_{3},x_{7},x_{8},x_{9},x_{11},x_{12}\}$
$\{x_{2}\}$	$\{x_{2},x_{4},x_{5},x_{6},x_{10}\}$	$\{x_{2},x_{10}\}$	$\{x_{2},x_{4},x_{6},x_{10}\}$	$\{x_{2},x_{4},x_{6},x_{10}\}$	$\{x_{2},x_{4},x_{5},x_{6},x_{8},x_{10},x_{12}\}$
$\{x_{3}\}$	$\{x_{1},x_{3},x_{7},x_{8}\}$	$\{x_{3},x_{7}\}$	$\{x_{3},x_{7}\}$	$\{x_{3},x_{7},x_{8}\}$	$\{x_{1},x_{3},x_{7},x_{8},x_{9},x_{11}\}$
$\{x_{4}\}$	$\{x_{2},x_{4},x_{6},x_{10},x_{12}\}$	$\{x_{4},x_{6}\}$	$\{x_{2},x_{4},x_{6},x_{10}\}$	$\{x_{2},x_{4},x_{6},x_{10}\}$	$U-\{x_{1},x_{3},x_{7},x_{8}\}$
$\{x_{5}\}$	$\{x_{2},x_{5},x_{8},x_{10},x_{12}\}$	$\{x_{5}\}$	$\{x_{5}\}$	$\{x_{5}\}$	$U-\{x_{1},x_{9}\}$
$\{x_{6}\}$	$\{x_{2},x_{4},x_{6},x_{10},x_{12}\}$	$\{x_{4},x_{6}\}$	$\{x_{2},x_{4},x_{6},x_{10}\}$	$\{x_{2},x_{4},x_{6},x_{10}\}$	$U-\{x_{1},x_{3},x_{7},x_{8}\}$
$\{x_{7}\}$	$\{x_{1},x_{3},x_{7},x_{8}\}$	$\{x_{3},x_{7}\}$	$\{x_{3},x_{7}\}$	$\{x_{3},x_{7},x_{8}\}$	$\{x_{1},x_{3},x_{5},x_{7},x_{8},x_{9},x_{11}\}$
$\{x_{8}\}$	$\{x_{3},x_{5},x_{7},x_{8}\}$	$\{x_{8}\}$	$\{x_{8}\}$	$\{x_{3},x_{7},x_{8}\}$	$U-\{x_{4},x_{6},x_{9},x_{11}\}$
$\{x_{9}\}$	$\{x_{1},x_{9},x_{11},x_{12}\}$	$\{x_{9},x_{11}\}$	$\{x_{9},x_{11}\}$	$\{x_{9},x_{11}\}$	$U-\{x_{2},x_{8},x_{10}\}$
$\{x_{10}\}$	$\{x_{2},x_{4},x_{5},x_{6},x_{10}\}$	$\{x_{2},x_{10}\}$	$\{x_{2},x_{4},x_{6},x_{10}\}$	$\{x_{2},x_{4},x_{6},x_{10}\}$	$\{x_{2},x_{4},x_{5},x_{6},x_{8},x_{10},x_{12}\}$
$\{x_{11}\}$	$\{x_{1},x_{9},x_{11},x_{12}\}$	$\{x_{9},x_{11}\}$	$\{x_{9},x_{11}\}$	$\{x_{9},x_{11}\}$	$U-\{x_{2},x_{8},x_{10}\}$
$\{x_{12}\}$	$\{x_{4},x_{5},x_{6},x_{9},x_{11},x_{12}\}$	$\{x_{12}\}$	$\{x_{12}\}$	$\{x_{12}\}$	$U-\{x_{3},x_{7}\}$

**Table 10:** Comparison of accuracy measures in $N_{1}$, $C_{1}$, $V_{1}^{0.8}$, $V_{1}^{0.75}$, and $E_{1}$-neighborhood approximation spaces in Example 5

Neighborhood	$V=\{x_{2},x_{4},x_{5},x_{6},x_{8},x_{10},x_{12}\}$			$W=\{x_{1},x_{3},x_{7},x_{8},x_{9}\}$		
	$\underline{B}$	$\overline{B}$	$\rho$	$\underline{B}$	$\overline{B}$	$\rho$
$N_{1}$	$\{x_{2},x_{4},x_{6},x_{10}\}$	$U-\{x_{1}\}$	$\dfrac{4}{11}$	$\{x_{3},x_{7}\}$	$U-\{x_{2},x_{4},x_{6},x_{10}\}$	$\dfrac{1}{4}$
$C_{1}$	*V*	*V*	1	$\{x_{1},x_{3},x_{7},x_{8}\}$	$\{x_{1},x_{3},x_{7},x_{8},x_{9},x_{11}\}$	$\dfrac{2}{3}$
$V_{1}^{0.8}$	*V*	*V*	1	$\{x_{1},x_{3},x_{7},x_{8}\}$	$\{x_{1},x_{3},x_{7},x_{8},x_{9},x_{11}\}$	$\dfrac{2}{3}$
$V_{1}^{0.75}$	$\{x_{2},x_{4},x_{5},x_{6},x_{10},x_{12}\}$	$U-\{x_{1},x_{3},x_{9},x_{11}\}$	$\dfrac{3}{4}$	$\{x_{1},x_{3},x_{7},x_{8}\}$	$\{x_{1},x_{3},x_{7},x_{8},x_{9},x_{11}\}$	$\dfrac{2}{3}$
$E_{1}$	$\{x_{2},x_{10}\}$	*U*	$\frac{1}{6}$	$\emptyset$	*U*	0

For comparison,

Table 10 shows the accuracy measures of the two sets $V=\{x_{2},x_{4},x_{5},x_{6},$ $x_{8},x_{10},x_{12}\}$ and $W=\{x_{1},x_{3},x_{7},x_{8},x_{9}\}$ with respect to all kinds of neighborhoods in Table 9. They satisfy $\rho _{E_{1}}(V)\le \rho _{N_{1}}(V)\le \rho _{V_{1}^{0.75}}(V)\le \rho _{V_{1}^{0.8}}(V)\le \rho _{C_{1}}(V)$ and $\rho _{E_{1}}(W)\le \rho _{N_{1}}(W)\le \rho _{V_{1}^{0.75}}(W)\le \rho _{V_{1}^{0.8}}(W)\le \rho _{C_{1}}(W)$.

These demonstrate that the accuracy measure of the proposed approximations based on $V_{j}^{\beta }$-neighborhood is higher than that based on $N_{j}$, $E_{j}$-neighborhoods, although it is not necessarily inferior to that based on $C_{j}$-neighborhoods. However, it can adjust the accuracy measure according to actual needs.

### Attribute reduction based on $V_{j}^{\beta }$-neighborhoods

It is well known that there is a lot of incompleteness in real data, especially when the data are high-dimensional large-scale data; it is particularly important to remove redundant attributes to reduce computing time and ensure the simplicity of knowledge acquisition. Therefore, this subsection proposes an attribute reduction algorithm based on attribute signification under $V_{j}^{\beta }$-neighborhood-based rough set models for incomplete neighborhood decision systems. First, we study the approximation precision of a subset in an incomplete information system.

For an information system $(U,{\varvec{C}}\cup {\varvec{D}}, V,f)$, where *U* is a nonempty and finite universe, ${\varvec{C}}$ is the set of condition attributes and ${\varvec{D}}$ is the set of decision attributes, $V=\{V_{a}\vert a\in {\varvec{C}}\cup {\varvec{D}}\}$ is the set of all attribute values and $f_{a}: U \rightarrow V_{a}$ is a function for any attribute *a*. If there exist some missing values $*$ in some attribute values, we say the system is an incomplete information system.

Considering that the types of attribute values in an information system are diverse, we must use different methods to construct the *N*-neighborhood of an element based on different types of attribute values.

For any $x\in U$ and any $a\in {\varvec{C}}$:

1. If $V_{a}$ is continuous, the *N*-neighborhood of *x* about the attribute *a* is defined as $N_{a}(x)=\{y\in U\mid \frac{\mid f_{a}(x)-f_{a}(y)\mid }{max\{V_{a}\}-min\{V_{a}\}}\le \delta \}$; here, $\delta$ is a given small positive number. In this paper, $\delta =0.5$.
2. If $V_{a}$ is discrete (including Boolean and symbolic), the *N*-neighborhood of *x* about *a* is defined as $N_{a}(x)=\{y\in U\vert f_{a}(x)=f_{a}(y)\}$.
3. If there are some missing values in the system, that is, $f_{a}(y)=*$, we think $y\in N_{a}(x)$.

Here, $N_{a}(x)$ can be regarded as the $N_{1}$-neighborhood of *x* in Definition 3. Since the neighborhoods are symmetric, $N_{a}(x)$ is also the $N_{2}$-neighborhood of *x*. Then, we deduce $N_{3}(x),\cdots ,N_{8}(x)$, $P_{j}(x)$, $C_{j}(x)$, $V_{j}^{\beta }(x)$ and $E_{j}(x)$ ($j=1,2,\cdots ,8$) based on Definition 5, 7, 9, and 14, respectively

.

Hence, the $N_{1}$-neighborhood system $N_{{\varvec{C}},1}(U)=\{N_{{\varvec{C}},1}(x)\vert x\in U\}$ in $(U,{\varvec{C}} \cup {\varvec{D}},V,f)$ follows:

1 $$\begin{aligned} N_{{\varvec{C}},1}(x)=\{y\in U\vert \forall a\in {\varvec{C}},~y\in N_{a}(x).\}. \end{aligned}$$

Then, we have $P_{{\varvec{C}},1}(U), C_{{\varvec{C}},1}(U), V_{{\varvec{C}},1}^{\beta }(U)$ and $E_{{\varvec{C}},1}(U)$.

It is worth noting that $N_{1}$-neighborhoods in Definition 3 may not satisfy the reflexivity based on arbitrary binary relation in general theory, while the $N_{1}$-neighborhoods in (1) are reflexive in the information systems.

#### Definition 19

Let $(U,{\varvec{C}} \cup {\varvec{D}},V,f)$ be an information system. For any $X\in U/{\varvec{D}}$

$$\begin{aligned} \eta _{V_{j}^{\beta }}(X)=\frac{\left| \underline{B_{V_{{\varvec{C}},j}^{\beta }}^{(1)}}(X)\right| }{\mid X\mid } \end{aligned}$$

is called the $V_{j}^{\beta }$-neighborhood local dependence measure of condition attribute sets ${\varvec{C}}$ with respect to decision class *X*. For any ${\varvec{B}}\subseteq {\varvec{D}}$

$$\begin{aligned} \gamma _{V_{j}^{\beta }}({\varvec{B}},{\varvec{D}})=\frac{\sum \limits _{X\in U/{\varvec{D}}}\left| \underline{B_{V_{{\varvec{B}},j}^{\beta }}^{(1)}}(X)\right| }{\sum \limits _{X\in U/{\varvec{D}}}\mid X\mid } \end{aligned}$$

is called the $V_{j}^{\beta }$-neighborhood total dependence measure of condition attribute subsets ${\varvec{B}}$ with respect to ${\varvec{D}}$. Here, $j=1,2,\cdots ,8$.

Similarly, we have the $N_{j}$, $P_{j}$, $C_{j}$, and $E_{j}$-neighborhood local and total dependence measures, denoted as

$$\begin{aligned} \eta _{N_{j}}(X)&=\frac{\left| \underline{B_{N_{{\varvec{C}},j}}}(X)\right| }{\mid X\mid },&\eta _{P_{j}}(X)&=\frac{\left| \underline{B_{P_{{\varvec{C}},j}}}(X)\right| }{\mid X\mid },\\ \eta _{C_{j}}(X)&=\frac{\left| \underline{B_{C_{{\varvec{C}},j}}}(X)\right| }{\mid X\mid },&\eta _{E_{j}}(X)&=\frac{\left| \underline{B_{E_{{\varvec{C}},j}}}(X)\right| }{\mid X\mid };\\ \gamma _{N_{j}}({\varvec{B}},{\varvec{D}})&=\frac{\sum \limits _{X\in U/{\varvec{D}}}\left| \underline{B_{N_{{\varvec{B}},j}}}(X)\right| }{\sum \limits _{X\in U/{\varvec{D}}}\mid X\mid },&\gamma _{P_{j}}({\varvec{B}},{\varvec{D}})&=\frac{\sum \limits _{X\in U/{\varvec{D}}}\left| \underline{B_{P_{{\varvec{B}},j}}}(X)\right| }{\sum \limits _{X\in U/{\varvec{D}}}\mid X\mid },\\ \gamma _{C_{j}}({\varvec{B}},{\varvec{D}})&=\frac{\sum \limits _{X\in U/{\varvec{D}}}\left| \underline{B_{C_{{\varvec{B}},j}}}(X)\right| }{\sum \limits _{X\in U/{\varvec{D}}}\mid X\mid },&\gamma _{E_{j}}({\varvec{B}},{\varvec{D}})&=\frac{\sum \limits _{X\in U/{\varvec{D}}}\left| \underline{B_{E_{{\varvec{B}},j}}}(X)\right| }{\sum \limits _{X\in U/{\varvec{D}}}\mid X\mid }. \end{aligned}$$

#### Example 6

We select the four incomplete data with different sizes from UCI (UCI Machine Learning Repository 2023) database listed in Table 11.

**Table 11:** Datasets description in Example 6

Datasets	Numbers of data	Numbers of attributes	Numbers of decisions
Chronic Kidney Disease	400	24	2
Annealing	798	38	5
LSVT Voice Rehabilitation	126	310	2
Obesity	1221	16	7

**Fig. 1:**
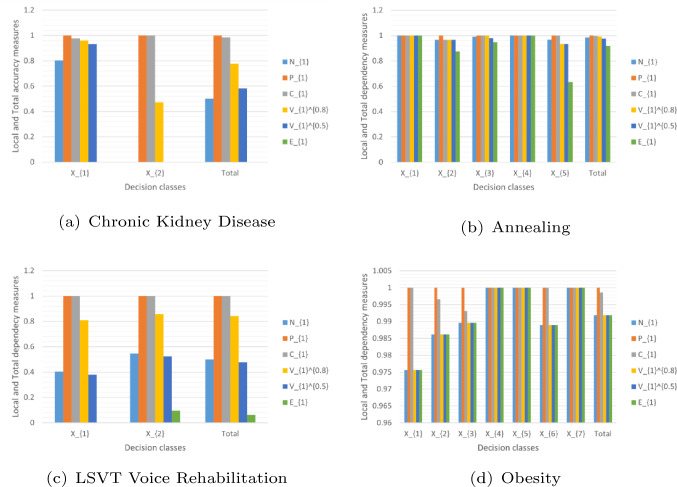
Local and total dependency measures in Example 6

**Fig. 2:**
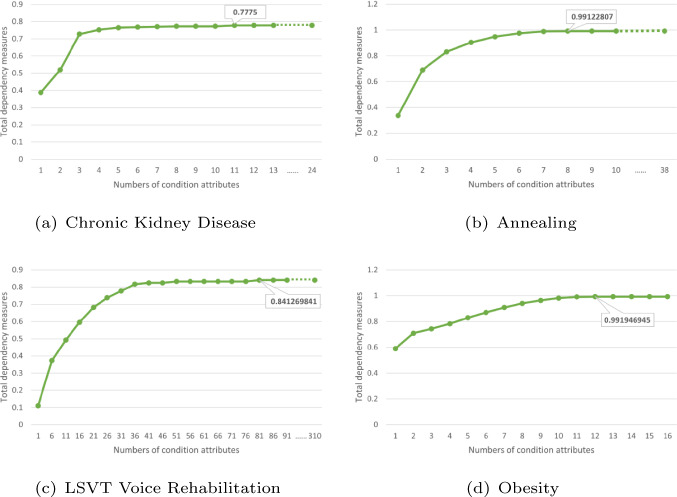
$\gamma _{V_{1}^{0.8}}$ of varying condition attributes in Example 6

We calculate the local dependency measures of the four data sets, including $\eta _{N_{1}}$, $\eta _{P_{1}}$, $\eta _{C_{1}}$, $\eta _{V_{1}^{0.8}}$, $\eta _{V_{1}^{0.5}}$, and $\eta _{E_{1}}$. And the total dependency measures, such as $\gamma _{N_{1}}({\varvec{C}},{\varvec{D}})$, $\gamma _{P_{1}}({\varvec{C}},{\varvec{D}})$, $\gamma _{C_{1}}({\varvec{C}},{\varvec{D}})$, $\gamma _{V_{1}^{0.8}}({\varvec{C}},{\varvec{D}})$, $\gamma _{V_{1}^{0.5}}({\varvec{C}},{\varvec{D}})$ and $\gamma _{E_{1}}({\varvec{C}},{\varvec{D}})$. See detailed in Fig. 1. It shows that for any data set, any $X_{i}\in U/{\varvec{D}}$

$$\begin{aligned} \eta _{P_{1}}(X_{i})\ge \eta _{C_{1}}(X_{i})\ge \eta _{V_{1}^{0.8}}(X_{i})\ge \eta _{V_{1}^{0.5}}(X_{i})\ge \eta _{E_{1}}(X_{i}), \end{aligned}$$

$$\begin{aligned} &\gamma _{P_{1}}({\varvec{C}},{\varvec{D}})\ge \gamma _{C_{1}}({\varvec{C}},{\varvec{D}})\ge \gamma _{V_{1}^{0.8}}({\varvec{C}},{\varvec{D}})\\&\,\,\,\,\ge \gamma _{V_{1}^{0.5}}({\varvec{C}},{\varvec{D}})\ge \gamma _{E_{1}}({\varvec{C}},{\varvec{D}}). \end{aligned}$$

The results again verify that the granularity of our proposed neighborhood system is between the coarse granularity based on the $C_{j}$-neighborhood system and the fine granularity based on the $E_{j}$-neighborhood system, and we can adjust the granularity size according to requirements.

At the same time, the dependency measures based on $N_{1}$-neighborhoods are not comparable to that based on $V_{1}^{0.8}$ or $V_{1}^{0.5}$-neighborhoods, while $\eta _{C_{1}}(X_{i})\ge \eta _{N_{1}}(X_{i})\ge \eta _{E_{1}}(X_{i})$ and $\gamma _{C_{1}}({\varvec{C}},{\varvec{D}})\ge \gamma _{N_{1}}({\varvec{C}},{\varvec{D}})\ge \gamma _{E_{1}}({\varvec{C}},{\varvec{D}})$.

Furthermore, we record the $V_{1}^{0.8}$-neighborhood total dependency measures $\gamma _{V_{1}^{0.8}}$ as a function of condition attributes in Fig. 2.

The four functions are monotonically increasing functions, so the total dependency measure can be seen as the index for attribute reduction.

#### Proposition 17

Let $(U,{\varvec{C}}\cup {\varvec{D}},V,f)$ be an information system and ${\varvec{B}}_{1}\subseteq {\varvec{B}}_{2}\subseteq {\varvec{C}}$. Then, $\gamma _{V_{j}^{\beta }}({\varvec{B}}_{1},{\varvec{D}})\le \gamma _{V_{j}^{\beta }}({\varvec{B}}_{2},{\varvec{D}})$ $(j=1,2,\cdots ,8)$.

#### Proof

Since ${\varvec{B}}_{1}\subseteq {\varvec{B}}_{2}$ and $V_{{\varvec{B}},j}^{\beta }(x)=\{y\in U\vert \forall a\in {\varvec{B}},~y\in V_{a,j}^{\beta }(x)\}$, $V_{\varvec{B_{1}},j}^{\beta }(x)\supseteq V_{{\varvec{B}}_{2},j}^{\beta }(x)$. Based on Definition 15, it is true $\underline{B_{V_{{\varvec{B}}_{1},j}^{\beta }}^{(1)}}(X)\subseteq \underline{B_{V_{{\varvec{B}}_{2},j}^{\beta }}^{(1)}}(X)$ for any $X\in U/{\varvec{D}}$. And, $\sum \limits _{X\in U/{\varvec{D}}}\mid X\mid =\mid U\mid$. Hence, $\gamma _{V_{j}^{\beta }}({\varvec{B}}_{1},{\varvec{D}})\le \gamma _{V_{j}^{\beta }}({\varvec{B}}_{2},{\varvec{D}})$. $\square$

#### Definition 20

Let $(U,{\varvec{C}}\cup {\varvec{D}},V,f)$ be an information system and ${\varvec{B}}\subseteq {\varvec{C}}$. If $\gamma _{V_{j}^{\beta }}({\varvec{B}},{\varvec{D}})=\gamma _{V_{j}^{\beta }}({\varvec{C}},{\varvec{D}})$ and $\gamma _{V_{j}^{\beta }}({\varvec{B}}',{\varvec{D}})\ne \gamma _{V_{j}^{\beta }}({\varvec{C}},{\varvec{D}})$ for any ${\varvec{B}}'\subset {\varvec{B}}$, then we say ${\varvec{B}}$ is a relative reduct with respect to ${\varvec{D}}$.

Similar to classical rough sets, there may exist more than one above relative reducts about ${\varvec{D}}$. The intersection of all the relative reducts about ${\varvec{D}}$ is called the relative core with respect to ${\varvec{D}}$. In some cases, the core may be empty. However, the relative reducts all have the same or better discriminative ability as the set containing all the condition attributes in the system. What is more, they are all indispensable.

#### Definition 21

Let $(U,{\varvec{C}}\cup {\varvec{D}},V,f)$ be an information system and ${\varvec{B}}\subseteq {\varvec{C}}$. For any $a\in {\varvec{C}}-{\varvec{B}}$, the outer significance measure of *a* with respect to ${\varvec{D}}$ is defined as

$$\begin{aligned} SIG(a,{\varvec{B}},{\varvec{D}})=\gamma _{V_{j}^{\beta }}({\varvec{B}}\cup \{a\},{\varvec{D}})-\gamma _{V_{j}^{\beta }}({\varvec{B}},{\varvec{D}}). \end{aligned}$$

The outer significance measure can show the importance of every attribute by a heuristic attribute reduction algorithm. The method of attribute reduction based on $V_{j}$-neighborhoods can avoid the case of empty $N_{j}$-neighborhoods, while it takes a little bit more time. The concrete algorithm is listed in Algorithm 1.
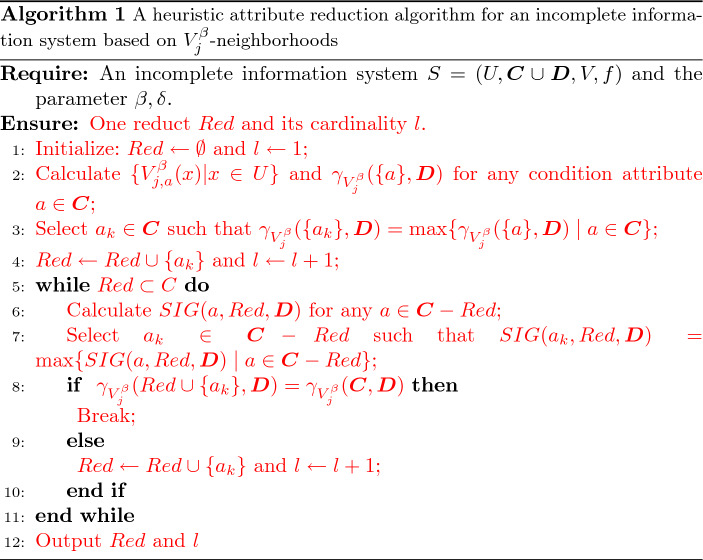


If the number of the objects is *n* and that of condition attributes is *m*, the computational complexity for $N_{1}$-neighborhood systems is $O(n^{2}m)$. The procedure for calculating $V_{1}^{\beta }$-neighborhood systems may need $O(n^{2})$ computation time. And the worst search for attribute reducts will bring about $(m^{2}+m)/2$ evaluations based on the significance measures. Hence, the overall time complexity of the algorithm may be $O(n^{2}(m+1)+(m^{2}+m)/2)$.

We apply the algorithm for the experiment analysis in Example 6. The results are shown in Table 12.

**Table 12:** Comparison of the numbers of attribute reductions with various neighborhood rough sets in Example 6

Data Sets	Numbers of attributes	Numbers of selected attributes					
		$N_{1}$	$P_{1}$	$C_{1}$	$V_{1}^{0.8}$	$V_{1}^{0.5}$	$E_{1}$
Chronic Kidney Disease	24	15	3	9	11	7	2
Annealing	38	9	7	9	8	10	6
LSVT Voice Rehabilitation	310	26	3	6	38	30	5
Obesity	16	12	4	14	12	12	12

It can be found from Table 12 that, in general, the finer the granularity of the neighborhood approximation spaces, the fewer selected attributes are obtained, such as $P_{1}$, $C_{1}$-neighborhoods; but if the granularity is too coarse, such as $E_{1}$-neighborhoods, the significance measure based on the neighborhoods may not be possible to discern the effects of different attributes, since there may be cases where the positive domain is an empty set (as shown in Fig. 1a and c).

In summary, the proposed $V_{j}^{\beta }$ neighborhood rough set not only can depict the incomplete information systems perfectly but also can adjust the granularity according to the needs of practical problems.

## Conclusions

In the real world, there is a huge amount of incomplete information that needs to be processed quickly and efficiently. Many researchers proposed various types of neighborhood systems and applied them to process imperfect knowledge. However, they may encounter empty neighborhoods or cannot handle incomplete information accurately.

This paper defines the eight types of variable precision neighborhoods based on inclusion degrees and introduces $V_{j}^{\beta }$-neighborhood-based rough set models. The properties and topology structures of the novel rough approximations are analyzed. The analysis results show that $V_{j}^{\beta }$-neighborhood-based rough set model extends $P_{j}$ and $C_{j}$-neighborhood-based ones by a predefined precision threshold $\beta$. Comparing with the existing $N_{j}$, $P_{j}$, $C_{j}$ and $E_{j}$-neighborhoods, the novel $V_{j}^{\beta }$-neighborhoods must be reflexive and can adjust the accuracy of approximation spaces according to the needs of the problems, which can more accurately and flexibly reflect incomplete information.

However, $V_{j}^{\beta }$-neighborhood-based rough set models still rely on a single binary relation and more risk-taking by handling continuous data sets, which may limit applications and lead to an increase in complexity. In the future, we will generalize the novel generalized rough sets to multi-granulation rough set models based on variable containment neighborhoods and extend fuzzy variable containment neighborhoods, and so on.

## Data Availability

The data used to support the findings of the work are included within the paper or originated from UCI database (http://archive.ics.uci.edu/ml/index.php).
